# Transcriptomics insights into the genetic regulation of root apical meristem exhaustion and determinate primary root growth in *Pachycereus pringlei* (Cactaceae)

**DOI:** 10.1038/s41598-018-26897-1

**Published:** 2018-06-04

**Authors:** Gustavo Rodriguez-Alonso, Marta Matvienko, Mayra L. López-Valle, Pedro E. Lázaro-Mixteco, Selene Napsucialy-Mendivil, Joseph G. Dubrovsky, Svetlana Shishkova

**Affiliations:** 1 Departamento de Biología Molecular de Plantas, Instituto de Biotecnología, Universidad Nacional Autónoma de México, Cuernavaca, Mexico; 2 Tecan Systems, 2450 Zanker Rd, San Jose, CA 95131 United States

## Abstract

Many Cactaceae species exhibit determinate growth of the primary root as a consequence of root apical meristem (RAM) exhaustion. The genetic regulation of this growth pattern is unknown. Here, we *de novo* assembled and annotated the root apex transcriptome of the *Pachycereus pringlei* primary root at three developmental stages, with active or exhausted RAM. The assembled transcriptome is robust and comprehensive, and was used to infer a transcriptional regulatory network of the primary root apex. Putative orthologues of *Arabidopsis* regulators of RAM maintenance, as well as putative lineage-specific transcripts were identified. The transcriptome revealed putative orthologues of most proteins involved in housekeeping processes, hormone signalling, and metabolic pathways. Our results suggest that specific transcriptional programs operate in the root apex at specific developmental time points. Moreover, the transcriptional state of the *P*. *pringlei* root apex as the RAM becomes exhausted is comparable to the transcriptional state of cells from the meristematic, elongation, and differentiation zones of *Arabidopsis* roots along the root axis. We suggest that the transcriptional program underlying the drought stress response is induced during Cactaceae root development, and that lineage-specific transcripts could contribute to RAM exhaustion in Cactaceae.

## Introduction

**Figure 1:**
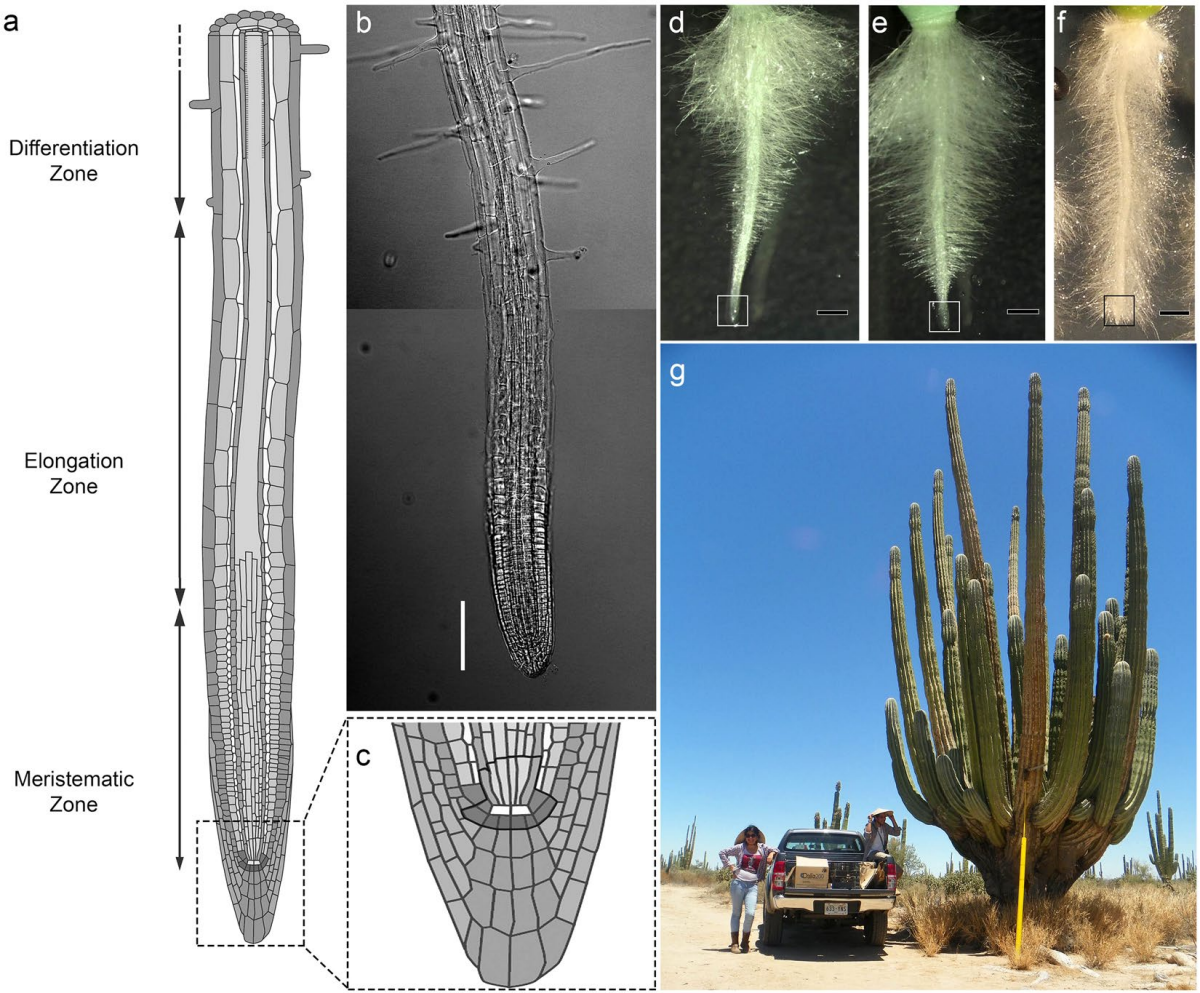
Indeterminate and determinate root growth. (**a**) Growing roots can be divided in three developmental zones along the longitudinal axis as illustrated here for the *Arabidopsis thaliana* primary root; the root apical meristem (RAM) is located in the meristematic zone and it is present and active in most angiosperm roots for long periods. (**b**) The developmental zones in an *A*. *thaliana* lateral root can be distinguished by the cell features. Scale bar: 100 µm. (**c**) The RAM contains a quiescent centre composed of cells with very low mitotic rate (white cells in the scheme). The cells adjacent to quiescent centre, delimited with a thick line, are called stem (initial) cells, and are a source of the dividing cells for the meristem. (**d**–**e**): The *Pachycereus pringlei* primary root exhibits determinate growth. In this work, 1 mm of the primary root apex, delimited with white or black box, was collected at three developmental stages: initial, when the RAM is present and fully active (**d**); intermediate, when the RAM is smaller and the differentiation zone is closer to the root apex (**e**); and terminal, when the RAM is exhausted and all the cells in the root, including those at the root apex, are differentiated (**f**). A mature *P*. *pringlei* is shown in (**g**). (**a**) and (**c**) were taken and modified from Peret, Benjamin (2017): doi:10.6084/m9.figshare.5143987.v4, originally deposited on FigShare as open access content under a CC BY 4.0 license.

Plant growth and organogenesis are postembryonic processes sustained by the presence and activity of meristems, which act as reservoirs of pluripotent cells. The root apical meristem (RAM) contains cells with a high mitotic rate, which supply cells for organ growth^1^. After few cell divisions in the RAM, also known as meristematic zone, the cells are displaced from the root apex and enter the elongation zone, where they undergo a longitudinal expansion. The generation and elongation of cells at the meristematic and elongation zone, respectively, further displace the cells into the differentiation zone, where the root cells acquire distintive features of particular root tissues (Fig. 1a–c). The RAM is present and active in most roots throughout much of the plant’s lifecycle in the majority of angiosperms, and it is therefore generally assumed that root growth can continue indefinitely as long as the environmental conditions are suitable^2^. A less common growth pattern for roots is the determinate growth, in which all cells at the root apex cease to divide and become differentiated as a consequence of RAM exhaustion (Fig. 1d–f). Determinate growth has been documented in a variety of angiosperm species, most remarkably in the proteoid lateral roots of Proteaceae species^3,4^; the adventitious roots of *Ficus pumila*^5^; and the lateral roots of *Zea mays*^6^, *Opuntia arenaria*, and *O*. *tunicata*^7^. Determinate growth of the primary root has only been reported for Cactaceae species from the Cactoideae and Opuntiodeae subfamilies, and *Ferocactus peninsulae*, *Stenocereus thurberi*, *S*. *gummosus*^8^, and *Pachycereus pringlei*^9^ were the first species in which determinate growth of the primary root was studied. This growth pattern has subsequently been observed in species from the seven tribes of the Cactoideae subfamily, namely, Cacteae, Pachycereeae, Cereeae, Trichocereeae, Notocacteae, Rhipsalideae^10^, and Hylocereeae^10,11^. Seedlings of all analysed species from the first six tribes always exhibit determinate growth of the primary root. Interestingly, some individuals of *Epiphyllum phyllanthus*, an epiphytic species from the Hylocereeae tribe, which inhabits mesic environments, exhibit determinate growth^11^, while other seedlings from the same species exhibit indeterminate growth^10^ of the primary root. Cactoideae species either thrive in arid or semi-arid environments, or experience water deficit by exposure to air, such as the epiphytic species of the Rhipsalideae and Hylocereeae tribes. Determinate growth of Cactaceae primary root could therefore represent an evolutionary adaptation to severe drought.

Root hair development as a consequence of early termination of root growth enables faster water uptake and favours seedling establishment; moreover, in *Stenocereus* species, RAM exhaustion induces lateral root initiation and development^8^. In all desert Cactaceae species studied so far, determinate growth can also be observed in at least the first- and second-order lateral roots. Cactaceae species exhibit determinate growth of the primary root independent of the growth condition^8^. These data, together with the incidence of RAM exhaustion in lateral roots and the determinate growth of roots regenerated from calli^12^, indicate that RAM exhaustion in Cactaceae species is a genetically regulated developmental program, rather than a response to environmental conditions. The molecular mechanisms underlying RAM exhaustion in the Cactaceae primary root are unknown, although suppression subtractive hybridization has been used to identify some of the differentially expressed genes between the *S*. *gummosus* primary root apex with functional and exhausted RAM^10^.

Our knowledge of the genetic regulation of RAM establishment and maintenance is mainly derived from studies of *Arabidopsis thaliana* (*Arabidopsis*) mutants with short root phenotypes. These studies led to the identification of three major genetic regulatory pathways. The most important pathway for RAM maintenance involves the PLETHORA (PLT) transcription factors, which belong to the AP2/ERF superfamily^13^. PLT proteins are partially redundant, their activities are dose-dependent, and they share a high number of target genes^14^. Four of the six *Arabidopsis* PLT proteins, PLT1, PLT2, PLT3, and PLT4 (also known as BBM), have similar expression patterns in the root apex, with expression maxima in the quiescent centre (QC), very high expression levels in the stem (initial) cells, strong expression in the rest of the RAM, lower levels of expression in the elongation zone, and low to no expression in the differentiation zone^15^. The primary root of the *plt1* mutant of *Arabidopsis* exhibits subtle alterations of the cell division pattern in the QC and root cap cells, while *plt1 plt2* double mutants develop a much shorter primary root that stops growing soon after germination due to RAM exhaustion, therefore exhibiting determinate growth^15^.

In addition to their well-characterized roles in the radial organization of the root, the GRAS transcription cofactors SCARECROW (SCR) and SHORT-ROOT (SHR) are also involved in RAM maintenance. *Arabidopsis scr* and *shr* loss-of-function mutants exhibit a shorter primary root compared with the wild type, as well as changes in QC identity^16,17^. As a result, the RAM becomes disorganized and is lost, causing determinate growth^18–20^. Another transcription factor specifically expressed in the QC and involved in stem cell homeostasis in the root is WUSCHEL-RELATED HOMEOBOX 5 (WOX5)^21,22^. In *Arabidopsis wox5* loss-of-function mutants the columella stem cells differentiate and the QC cells are larger than those of the wild type, while the inducible ectopic expression of *WOX5* prevents differentiation of columella and lateral root cap cells^21,23^. When combined with *scr*, *shr*, or *plt1plt2* mutations, the *wox5* mutation redundantly accelerates RAM exhaustion^21^.

The exploration of RAM exhaustion and other developmental processes in Cactaceae has been limited by the lack of reference genomes for these species, although, after completion of this work, early draft genomes of four columnar cacti were reported^24^. Nevertheless, the rapid improvement of RNA-seq as a high-throughput sequencing technology enables the *de novo* assembly of transcriptomes for non-model species, circumventing the need for a reference genome^25^. In this study, we used RNA-seq to generate a *de novo* assembled transcriptome of the *P*. *pringlei* primary root apex at three developmental stages. To explore gene conservation in Cactaceae species, this transcriptome was compared with recently published *Lophophora williamsii* transcriptome^26^. The *P*. *pringlei* root apex transcriptome was used to assess differential gene expression and to infer a transcriptional regulatory network, with the aim of expanding our knowledge of RAM exhaustion in Cactaceae species and, consequently, enhancing our understanding of RAM maintenance in angiosperms.

## Results and Discussion

### Transcriptome *de novo* assembly and annotation

To examine the molecular mechanisms underpinning RAM exhaustion in Cactaceae, we *de novo* assembled the root apex transcriptome of the *P*. *pringlei* primary root at three developmental stages: i) initial, when the RAM was present, at around 1–4 days after germination (d.a.g.); ii) intermediate, when a smaller RAM was still present and the root hairs were closer to the root apex, at approximately 5–6 d.a.g.; and iii) terminal, when the RAM was exhausted and all cells of the root apex were differentiated, at 9–13 d.a.g. (Fig. 1d–f). RNA-seq of the six samples, two biological replicates per developmental stage, yielded 279.23 million 2 × 100-bp paired-end reads with an average of 46.5 million reads per sample. The processed reads (see Methods) from all samples were used to *de novo* assemble the *P*. *pringlei* root apex transcriptome. The transcriptome was assembled in two consecutive rounds, first with all the processed reads and mapping reads back to contigs. The unmapped reads were then used to assemble additional contigs under the same parameters of bubble- and word-size as the previous run. This approach allowed the recovery of highly similar sequences, which were expected for large gene families and allelic variants, as *P*. *pringlei* is a tetraploid species^27^. The assembled transcriptome included 49,045 contigs, hereafter referred as transcripts, of ≥400 nt, with an average length of 1,080 nt (Table 1). Transcripts ranging from 400 to 2,000 nt in length account for 87.63% of the total assembled sequences, while 10.94% and 1.43% of the total transcripts were 2,000–4,000 nt and >4,000 nt long, respectively.

**Table 1:** *De novo* assembled transcriptome of the *Pachycereus pringlei* primary root apex.

Sequencing and preprocessing		Assembly		Contig features	
Total reads	279.23 × 10^6^	Total contigs	49,045	Average length (nt)	1,080
Av. reads per sample	46.5 × 10^6^	N25	1,332	Max. length (nt)	14,583
Paired-end reads	79 × 10^6^	N50	728	Min. length (nt)	400
Merged reads	88 × 10^6^	N75	611	400–2,000 nt	87.63%
				2,000–4,000 nt	10.94%
				>4,000 nt	1.43%

**Figure 2:**
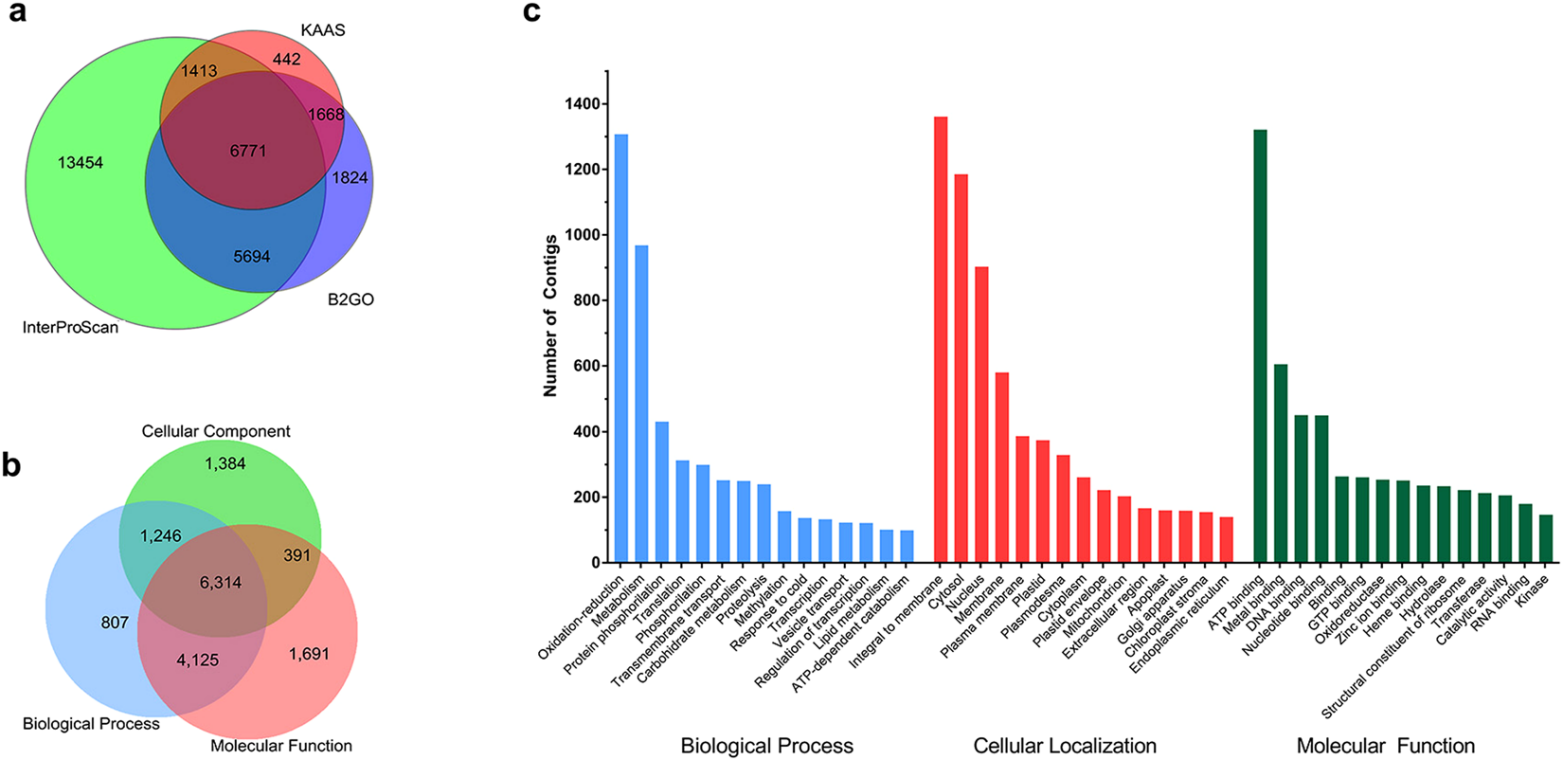
Annotation of the *de novo* assembled root tip transcriptome of *Pachycereus pringlei*. (**a**) 63.7% of the total assembled contigs were annotated using the KEGG Automatic Annotation Server (KAAS), InterProScan, or Blast2GO tools. (**b**) The number of contigs annotated per B2GO category. (**c**) The most frequently represented gene ontology subcategories.

A total of 31,265 contigs (63.7%) were annotated using the CLC GW BLAST2GO plug-in, InterProScan, or KAAS^28^ tools (Fig. 2). The species from which the highest number of significant hits was retrieved was *Beta vulgaris* (see Supplementary Fig. S1). This was expected as both, *B*. *vulgaris* and *P*. *pringlei*, belong to the order Caryophyllales and the former species was the closest relative of *P*. *pringlei* with an available reference genome^29^ at the time of the analysis. Shortly after this study, the genomes of the Caryophyllales *Amaranthus hypocondriacus*^30^ and *Chenopodium quinoa*^31^ were released; however, they were not available from the RefSeq database and therefore could not be included in the annotation analysis. The results of the KAAS annotation showed that the assembled transcriptome contained all the putative orthologue sequences required to reconstruct the central metabolism and CAM carbon fixation pathways (see Supplementary Fig. S2). Furthermore, the root apex transcriptome contains putative orthologues of most genes associated with the following: plant hormone signalling pathways, including auxin, cytokinin, gibberellin, abscisic acid, ethylene, brassinosteroid, jasmonic acid, and salicylic acid; eukaryotic DNA replication; basal eukaryotic transcription factors; synthesis, surveillance, and degradation of mRNA; ribosome biogenesis; and protein processing in the endoplasmic reticulum (Supplementary Fig. S2). Thus, the assembled root apex transcriptome is not only robust but also comprehensive and represents a step forward in the study of the Cactaceae family, for which the available transcriptomes generated so far include expressed sequence tags^32^ or specific RNA-seq datasets exploring metabolic processes^26,33^. The transcriptomes previously available for other plant species from the order Caryophyllales were also limited, but were recently expanded by the efforts of the OneKP initiative^34^.

### Transcriptional dynamics of the Cactaceae root development

**Figure 3:**
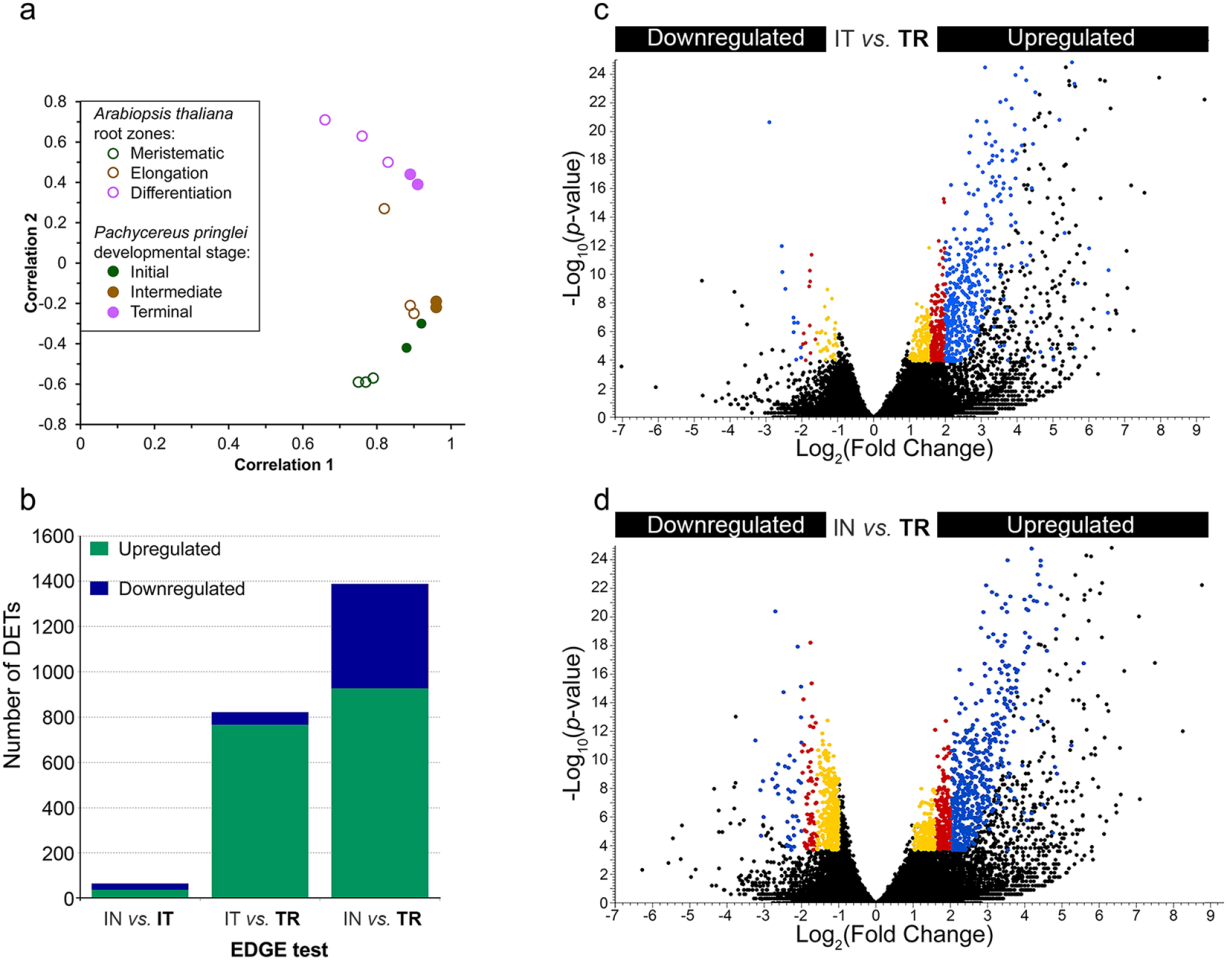
Characterization of the *Pachycereus pringlei* transcriptome. (**a**) Principal component analysis of *P*. *pringlei* developmental stages and *Arabidopsis* root zones and (**b**–**d**) the empirical detection of gene expression shows that the transcriptional state of the *P*. *pringlei* root tip changes during development. Only 66 differentially expressed transcripts (DETs) were identified in the comparison of the Initial *vs* Intermediate developmental stages (**b**), while 873 and 1,388 DETs were identified between the Intermediate *vs* Terminal (**b**,**c**), and Initial *vs* Terminal (**b**,**d**) stages. Colors in c and d correspond to the fold change (FC): blue, FC > 4; red, 3 < FC < 4; yellow, 2 < FC < 3. In all cases, the FDR *p*-value was ≤0.005. IN: Initial stage; IT: Intermediate stage; TR: Terminal stage. Upregulated and downregulated refer to the expression level at the developmental stage shown in bold.

To assess transcript abundance, the assembled transcripts were used as reference sequences to separately map the reads from each sample. Then, a principal component analysis (PCA) was performed on the correlation matrix to evaluate sample variability, and to visualize the transcriptional dynamics of the *P*. *pringlei* root apex at different developmental stages. The PCA analysis showed that the first and second principal components explain 84% and 12% of the observed variability of the data, respectively (see Supplementary Fig. S3). As shown in Fig. 3a, the PCA distinguished the terminal developmental stage transcriptomes from those of the initial and intermediate developmental stages. This was likely because all cells of the primary root apex are differentiated at the terminal developmental stage, while both, meristematic and elongating cells are present at the initial and intermediate stage, although in different proportions. The intermediate stage samples exhibited the least variability, as appreciated by their proximity to each other on the PCA plot, while the samples from the initial stage exhibited the greatest variability. This could be explained because the samples of the initial growth stage include root tips from 1–4 d.a.g. seedlings, resulting in the most heterogeneous biological replicates used in this study. The separation of the root apex samples according to the developmental stage, however, indicates that the observed variability between samples is due to transcriptional state changes during primary root development, and that specific transcriptional programs operate in the *P*. *pringlei* root apex at particular developmental time points. In contrast, in most angiosperms the RAM maintenance programs remain present in the root apex of the majority of roots.

To compare the transcriptional states of the *P*. *pringlei* root apex with those of *Arabidopsis*, the raw RNA-seq reads from the meristematic, elongation, and differentiation root zones of *Arabidopsis*^35^ were used. A PCA was performed on the *Arabidopsis* data and the results were visualized on the same plot as the *P*. *pringlei* data (Fig. 3a). The transcriptomes from the *P*. *pringlei* intermediate and terminal developmental stages were found to group with the transcriptomes of the *Arabidopsis* elongation and differentiation root zones, respectively. The initial stage transcriptome was positioned between those of the meristematic and elongation root zones, probably because the *P*. *pringlei* root apex in this stage includes cells from both the meristematic and elongation zones. The PCA results also suggest that the differentiated cells of the *P*. *pringlei* root apex are functional after RAM exhaustion, and that they could perform the typical functions of cells from the differentiation zone of other angiosperm roots. This observation is in congruence with our previous results, which indicated that RAM exhaustion in *S*. *gummosus* and *P*. *pringlei* does not involve programmed cell death^36^. The slight shift of the transcriptome from the *P*. *pringlei* terminal stage towards those of the *P*. *pringlei* intermediate stage and *Arabidopsis* elongation zone suggests that the *P*. *pringlei* root apex cells retain some expression programs from their previous, less differentiated state; however, this needs to be further explored. Together, these results indicate that cell differentiation along the primary root axis occurs at two different regions during *P*. *pringlei* primary root development: distal from the RAM, as in other angiosperms, but also at the very root apex as the RAM becomes exhausted.

### Differential gene expression

An extraction of differential gene expression (EDGE) test was performed to identify differentially expressed transcripts (DETs) in the root apex across developmental stages^37^. Transcripts were considered to be DETs when they met these criteria: fold change ≥2, FDR *p*-value≤ 0.005, and RPKM average value >3 at each of the developmental stages under comparison. As shown in Fig. 3b, with this stringency level, only 66 DETs were identified when comparing the Initial *vs*. Intermediate stage transcriptomes. By contrast, the numbers of DETs between the Intermediate *vs*. Terminal and Initial *vs*. Terminal stage transcriptomes were 873 and 1,388, respectively (Fig. 3b–d). Therefore, the number of DETs increased with the progression of root development. Since very few DETs were detected between the initial and intermediate stages, only the last two comparisons are shown in Fig. 3c,d. For both the Intermediate *vs*. Terminal and Initial *vs*. Terminal comparisons, the number of upregulated transcripts at the terminal stage was higher than the number of downregulated transcripts, with approximately 14 times and 2 times more upregulated transcripts, respectively (Fig. 3b). Hence, during the transition from a population of mainly elongating to differentiated cells, many genes are upregulated, while downregulation of expression is observed in far fewer genes. It is generally assumed that in fully differentiated cells, more genes are upregulated in comparison to undifferentiated or partially differentiated cells. This assumption is supported by data retrieved from Huang and Schiefelbein^35^ (see Supplementary Table S1). Surprisingly, among the DETs between the initial and terminal developmental stages, the number of upregulated genes was only twice that of downregulated genes.

**Figure 4:**
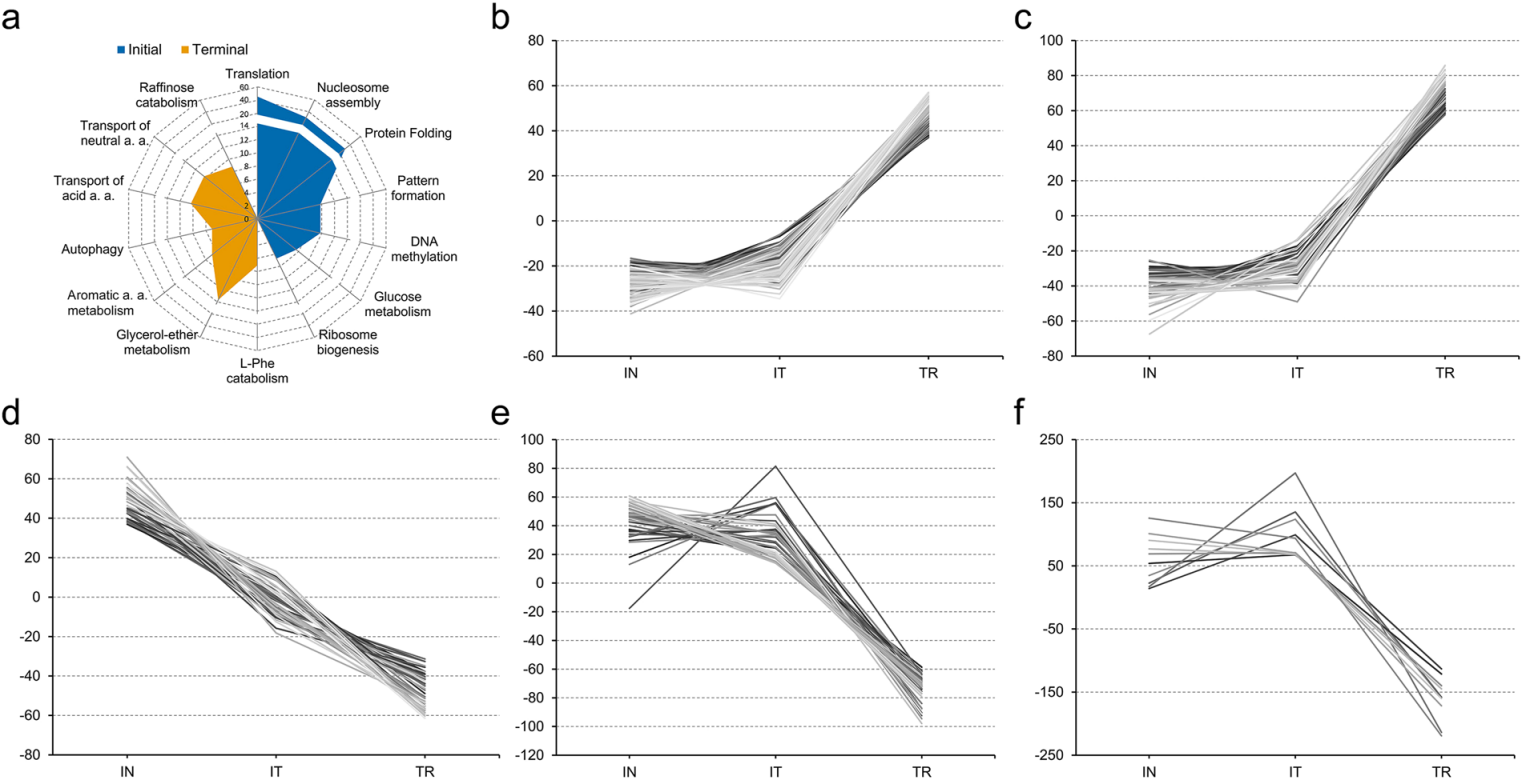
Expression profiles of the differentially expressed transcripts in the *Pachycereus pringlei* root apex. (**a**) BLAST2GO biological processes overrepresented in the Initial and Terminal developmental stages of the primary root apex of *P*. *pringlei*. Axis: -log_10_(*p*-val) of the enrichment test. (**b**–**f**) Representative expression profiles. Some of the functionally related transcripts from distinct GO categories could be grouped within a number of expression profiles. For example, several transcripts from the autophagy or amino acid transport GO categories, among others, are included in (**b**); the amino acid metabolism, amino acid transport, and glycerol-ether metabolism categories are included in (**c**); nucleosome assembly, translation, and regulation of DNA methylation, are included in (**d**); protein folding, protein deubiquitylation, and rRNA processing, are included in (**e**); some transcripts from the translation, and response to oxidative stress categories are included in (**f**). (**b**–**f**): Numbers on the y-axes correspond to the normalized and centered RPKM values for the Initial (IN), intermediate (IT), and Terminal (TR) growth stages.

A hypergeometric test^38^ based on the gene ontology (GO) biological process annotation was performed for the DETs. The results, shown in Fig. 4a, suggest that as the root apex cells transition from meristematic to fully differentiated cell states, processes such as protein synthesis and folding, DNA methylation, nucleosome assembly, and ribosome biogenesis are downregulated. By contrast, overrepresented GO subcategories at the terminal developmental stage include amino acid transport; metabolism of aromatic compounds and glycerol ether; autophagy; and stress responses. Furthermore, the DETs were grouped according to their RPKM values using the *k*-mean clustering method^39^. A total of 27 clusters were obtained, which can be grouped into three main expression profiles as shown in Fig. 4b–f: transcripts upregulated at the terminal developmental stage (Fig. 4b–c); transcripts downregulated at the terminal stage (Fig. 4d–e); and one cluster of transcripts upregulated at the intermediate developmental stage (Fig. 4f; the complete set of clusters is depicted in Supplementary Fig. S4). Clustering analysis is useful to identify sets of genes that could be involved in the same biological process, or influenced by the same transcriptional regulator. Remarkably, several transcripts predicted to encode 60 S or 40 S ribosomal proteins were grouped in the same cluster and were downregulated as root development progressed and the root apex cells differentiated. Among the transcripts downregulated at the terminal developmental stage, those encoding putative histones, histone deacetylases, some peroxidases, and gibberellin-regulated proteins (for example, *GASA1* and *GASA4/6* putative orthologues) were retrieved. Among the transcripts upregulated at the terminal developmental stage, there was an overrepresentation of abscisic acid induced transcripts, encoding members of the abscisic stress ripening protein family and abscisic acid receptors. This result suggests that the genetic programs to cope with drought stress are induced in the differentiated cells of the Cactaceae root in any growth condition, as the plants used for the transcriptome analyses were cultivated in Petri dishes and did not experience water deficit. Other DETs upregulated in the terminal developmental stage included transcripts encoding glutathione transferases; proline-rich cell wall proteins; ethylene-responsive, NAC-domain, and WRKY transcription factors; and proteins involved in auxin-related processes, such as ARFs and SAURs.

Remarkably, 17.4% (244) of the DETs could not be annotated and may represent lineage-specific transcripts. Moreover, a significant number of the DETs with a higher abundance in the terminal stage were annotated as uncharacterized proteins (7%) or hypothetical proteins (~2%), with significant hits in a variety of species, including *Populus trichocarpa* and *Phaseolus vulgaris*.

### Conservation of Cactaceae genes and identification of lineage specific transcripts

To evaluate the conservation of genes in the Cactaceae, we used the previously reported transcriptome of *L*. *williamsii*^26^. The *L*. *williamsii* shoot + root transcriptome consists of 40,436 unigenes ranging in length from 200 to 4,170 nt, which were compared with the *P*. *pringlei* root tip transcriptome (Supplementary Table S2, Supplementary Fig. S5). A high degree of conservation was revealed among the nucleotide sequences of both *de novo* assembled transcriptomes (Supplementary Table S3), with a median E-value of 2.25 × 10^-118^. Multiple hits from *L*. *williamsii* were retrieved for most *P*. *pringlei* query sequences, as the *P*. *pringlei* contigs were significantly longer (Supplementary Fig. S5; Supplementary Tables S2 and S3).

Next, to explore whether some of the 244 unannotated *P*. *pringlei* DETs in the Initial *vs* Terminal comparison could represent Cactaceae-specific transcripts, we performed a BLASTn search of these DETs against the *L*. *williamsii* unigenes, revealing significant hits for 53% (130) of them. Furthermore, significant hits for some of the unannotated DETs were also identified in the shoot cambial zone transcriptomes of other Cactaceae species, namely, *Ariocarpus retusus*, *Echinocactus platyacanthus*, *Ferocactus pilosus*, and *Pereskia lychnidiflora* (E. Petrone and T. Terrazas, pers. comm.). Of the DETs that could not be annotated, 163 (66%) contain ORFs encoding putative peptides longer than 50 aa. Of these, 10, 45, and 108 putative peptides were predicted to be >200 aa, 100–200 aa, and 50–100 aa long, respectively (Supplementary Fig. S6). These results strongly suggest the existence of lineage-specific transcripts and their possible involvement in the regulation of RAM exhaustion and determinate root growth in Cactaceae. This assumption requires further exploration as it could lead to the identification of novel root development regulatory molecules, opening up new opportunities for evo-devo research.

### RT-qPCR validation of expression profiles

**Figure 5:**
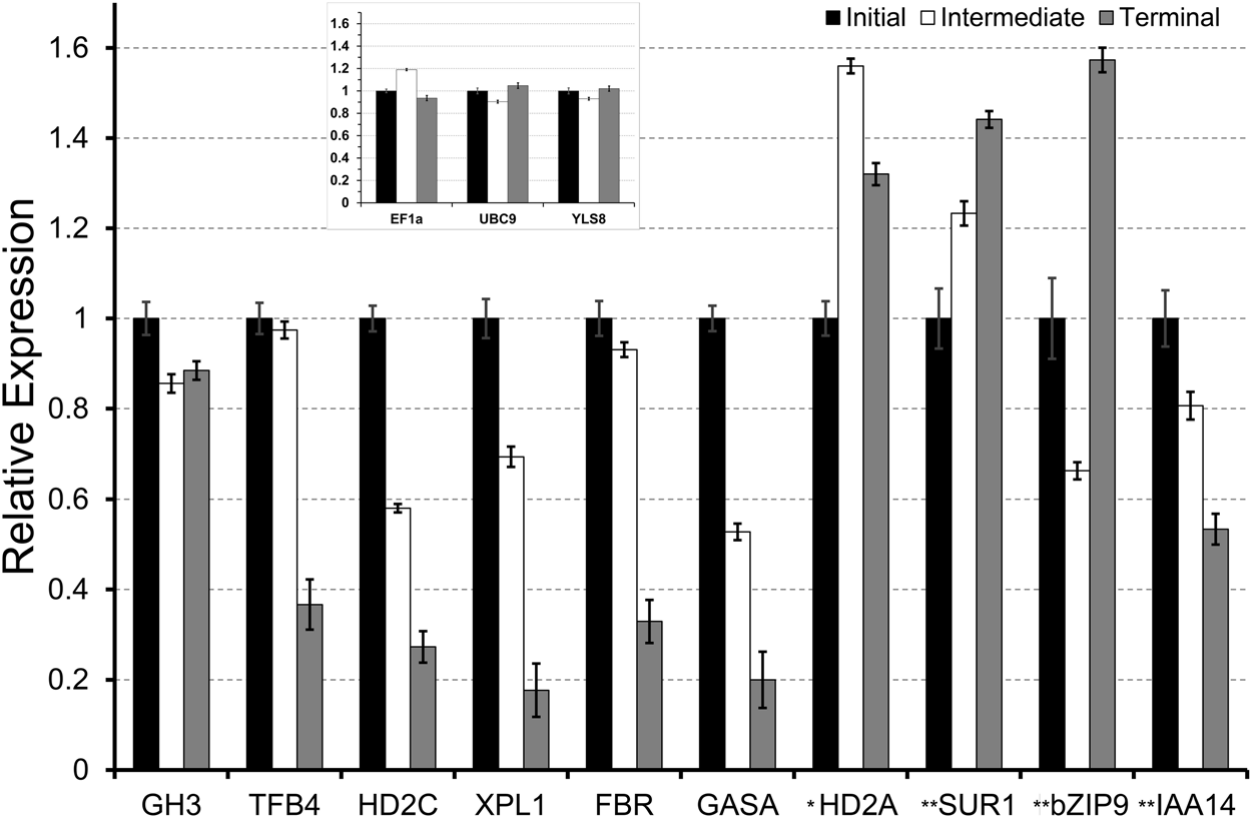
The relative expression levels of selected *Pachycereus pringlei* transcripts quantified using RT-qPCR assays for the primary root apex at the three stages of primary root development. The expression levels were normalized relative to *PpEF1α*, *PpUBC9*, and *PpYLS8*, respectively (inset). ^*^The trend for RPKM values and transcript abundance evaluated by qRT-PCR between initial and terminal development stages was different for this transcript. ^**^Two biological replicates were used for these three genes, and three biological replicates were used for another ten genes.

In our previous work, several DETs downregulated at the terminal growth stage in *S*. *gummosus* primary root apices were identified by suppression subtractive hybridization^10^ and confirmed using RT-qPCR. We therefore used RT-qPCR to measure the relative expression of putative orthologues of these DETs in *P*. *pringlei* root tips. First, a set of candidate reference genes was selected by identifying the putative *P*. *pringlei* orthologues of the *Arabidopsis* superior reference genes^40^. The list was reduced by selecting the *P*. *pringlei* transcripts with the lowest RPKM variation in the root apex at the initial, intermediate, and terminal stages of primary root development (see Supplementary Table S4, Supplementary Fig. S7). *PpEF1α*, *PpUBC9*, and *PpYLS8*, were selected as internal references. The expression patterns of *PpTFB4*, *PpHD2C*, *PpXPL*, *PpFIBR*, *PpGASA*, were evaluated and the downregulation in the *P*. *pringlei* root apex at the terminal stage was confirmed by transcriptome and RT-qPCR analyses (Fig. 5, Supplementary Fig. S8). This suggests that the mechanisms of determinate root growth are conserved at least within the Pachycereeae tribe of the subfamily Cactoideae. Furthermore, RT-qPCR analysis confirmed the trend in expression level throughout root development of seven more genes, including the three selected reference genes; *PpGH3*, a stably expressed transcript across root development; as well as, *PpIAA14*, downregulated at the terminal developmental stage; *PpbZIP9*, and *PpSUR1* upregulated at the terminal developmental stage. With the exception of *PpHD2A*, the expression profiles of 12 of the 13 tested genes were qualitatively reproduced using RT-qPCR, therefore validating the reliability of the *P*. *pringlei* RNA-seq (Fig. 5, Supplementary Fig. S8).

### Transcriptional regulatory network for the *P*. *pringlei* RAM

**Figure 6:**
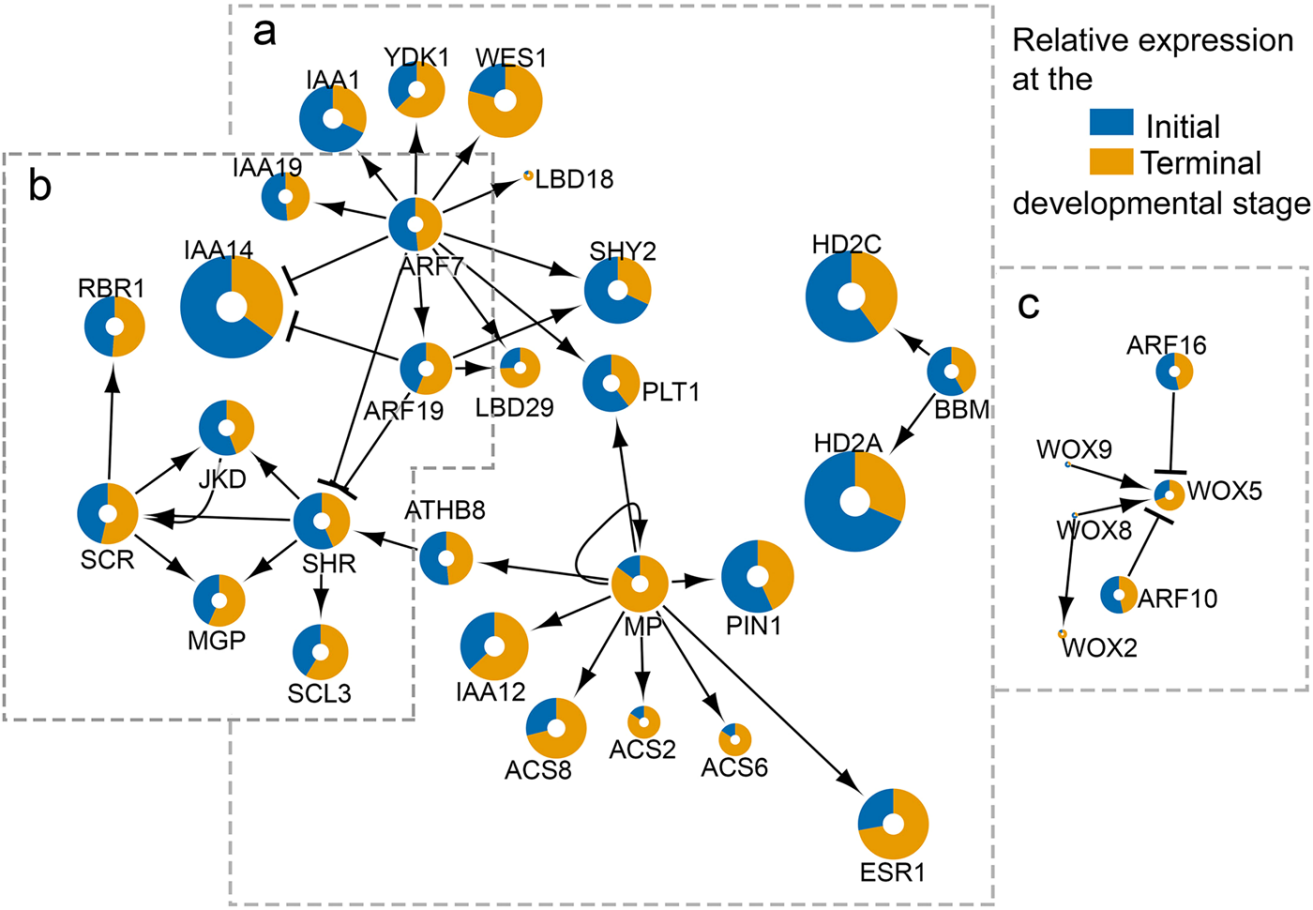
Root development modules of the *Pachycereus pringlei* inferred transcriptional regulatory network. The complete network (Fig. S9) was separated into three distinct modules: (**a**) PpPLT, which includes PpPLT1 and PpBBM as separate clusters; (**b**) PpSHR-PpSCR; and (**c**) PpWOX. Node size represents the average expression level (log_2_(RPKM)) of the contig in the *P*. *pringlei* transcriptome.

Cellular processes, either during development or in response to environmental stimuli, are not controlled by single genes, but by an intricate network connecting multiple elements, including but not limited to transcription factors and their target genes^41^. We therefore generated a transcriptional regulatory network (TRN) by retrieving a set of genes that were reported as important regulators of root development^35,42^. These genes and their first neighbours, together with their interactions, were extracted from the *Arabidopsis* transcriptional regulatory map^43^. The putative *P*. *pringlei* orthologues of these genes were identified by tBLASTn using best bidirectional hit (BBH) analysis. An interaction table for the retrieved orthologous *P*. *pringlei* nodes, and a table of properties for the *P*. *pringlei* nodes were generated based on the interactions of the *Arabidopsis* network (Supplementary Table S5, Supplementary Table S6). The resulting TRN inferred for the *P*. *pringlei* root apex included 81 nodes and 111 edges (see Supplementary Fig. S9). From these, 48 nodes corresponded to *Arabidopsis*-*P*. *pringlei* BBH, while 30 nodes were retrieved as unidirectional hits. No *P*. *pringlei* orthologues were identified for three *Arabidopsis* nodes, *YAB1*, *FEZ*, and *LFY*, probably due to their low or null expression levels in the *P*. *pringlei* root apex. The network was divided into the subnetworks shown in Fig. 6, which included orthologues of proteins involved in the three main *Arabidopsis* RAM regulatory pathways. The *PpPLT* subnetwork (Fig. 6a) was represented by *PpPLT1* and *PpBBM*, which were included in a module that also contained their interaction partners, including ARF proteins and histone deacetylases. At the time of the analysis, only these two PLT members were included in the *Arabidopsis* regulatory map. The recent identification of the target genes of distinct PLTs^14^ will provide valuable insight into the intricate regulation of RAM maintenance by these transcription factors, and would allow the inclusion of additional nodes and interactions to the *P*. *pringlei* TRN. The second subnetwork included the GRAS-domain transcription cofactors PpSHR and PpSCR (Fig. 6b), as well as the transcription factor PpJKD. Recent reports show that SHR, SCR, and JKD form a macromolecular complex that regulates the transcription of different genes according to their cellular context^44,45^; therefore, the apparent expression stability of these nodes in the *P*. *pringlei* root apex during root development might be explained by their involvement in developmental processes, such as radial pattern formation^16^, vascular differentiation^46^, and transition from cell division to cell elongation^47^, in addition to their roles in RAM maintenance. The *PpSHR* and *PpSCR* subnetwork also included other interactors, such as *PpMGP*, and *PpRBR* orthologues of *Arabidopsis* genes that have been demonstrated to be important regulators of RAM maintenance^48,49^. The third subnetwork was composed of genes encoding four WOX proteins and their ARF interacting partners (Fig. 6c). However, the expression level of the putative *WOX* orthologues was very low, with average expression levels <4 RPKM across the developmental stages of the *P*. *pringlei* root apex. The low RPKM values obtained for *PpWOX* are expected: for example, the *Arabidopsis WOX5* is expressed specifically in the QC cells^21^, while the samples for RNA-seq data presented here included a broader cell population.

In conclusion, our results demonstrate that the transcriptional programs operating in the *P*. *pringlei* root apex are similar to those of the *Arabidopsis* root zones. The described differences in expression level of RAM regulators in the root apex of the cactus across developmental stages suggest that the acquisition of the determinate growth as an adaptive trait might have arose as a result of changes in regulatory sequences. This assumption requires further exploration, which will be possible upon the improvement of the assembly and annotation of the recently published draft nuclear genomes of Pachycereeae tribe species, with a high-coverage genome of *Carnegiea gigantea* and low-coverage genome of three more columnar cacti, including *P*. *pringlei*^24^.

## Materials and Methods

### Plant materials

Fruit of *Pachycereus pringlei* (S. Watson) Britton & Rose were collected from plants (Fig. 1g) growing near Bahía Kino, Sonoran Desert, Mexico. The seeds were sterilized as described^36^ and germinated on vertically oriented Petri dishes containing 0.2 × Linsmaier and Skoog medium (Phyto Technology Laboratories, Lenexa, KS, USA; pH 5.8), 0.8% Bacto Agar (BD Difco, Sparks, MD, USA), with no sucrose added. Plants were grown at 28 °C in a 12/12-h photoperiod with a light intensity of 80 µmol m^-2^ s^-1^.

For the primary root apex samples, 1 mm sections of the root tip were detached using a sterilized razor blade and frozen in liquid nitrogen. Root apices at three developmental stages were collected: initial, intermediate, and terminal (Fig. 1d–f). Root hairs, formed by differentiated epidermal cells, served as a hallmark for cell differentiation to distinguish between root developmental stages. Only the apices of roots that grew in contact with the surface of the medium were used for RNA extraction; roots that grew inside the medium, or away from it, were not sampled. Two biological replicates were used for RNA-seq, and two or three additional biological replicates were used for RT-qPCR analysis. Approximately 350, 450, and 600 root apices were collected for each biological replicate of the initial, intermediate, and terminal developmental stages, respectively. Plant material was stored at -70 °C until RNA extraction.

### RNA extraction, RNA-seq, and *de novo* assembly

Total RNA was isolated using TRIzol reagent (Thermo-Fisher Scientific, Waltham, MA, USA). All steps of RNA quality analysis, RNA processing, library preparation and 100 cycles of paired-end sequencing were performed at BGI-Tech, Hong Kong. Illumina RNA Library Prep kit was used for cDNA synthesis and Illumina HiSeq 2000 platform was used for sequencing. The quality of the Illumina sequence reads was analysed with CLC Genomics Workbench v 7.5 (CLC GW, CLC bio, Qiagen, Hilden, Germany; http://www.clcbio.com/). Read processing was performed using CLC GW as follows: the terminal 13 nt of the 5′ end were trimmed, and if both reads from a single RNA fragment overlapped, they were merged. A quality and adaptor trimming step was run on merged and unmerged reads; resulting reads shorter than 40 nt were discarded. The root apex transcriptome assembly was performed several times using the CLC GW *de novo* assembly tool, setting 400 nt as the minimal value for contig length, and varying the word size and bubble size to achieve optimal values, which were 50 and 400 nt, respectively. Unassembled reads were used to perform a second round of assembly using the optimal bubble and word sizes from the previous assembly run. Contigs larger than 3,000 nt were extended using the Genome Finishing Module in CLC GW. Raw reads and assembled contigs were deposited in the NCBI Gene Expression Omnibus under the accession number GSE104832.

### Contig annotation and RNA-seq analysis

Contig (transcript) sequences were annotated using the CLC GW Blast2GO plugin, the InterPro database, and the KEGG Automated Annotation Service (KAAS^28^). The processed reads obtained for each developmental stage were independently mapped to the contigs generated in the *de novo* assembly, counted, and normalized using the reads per kilobase per million mapped reads (RPKM) method^50^. Principal component analysis (PCA) was performed on the correlation matrix with the RPKM count table for each developmental stage of *P*. *pringlei* and, separately, for each zone of *Arabidopsis* root^35^. Differential expression was assessed with EDGE test^37^ as implemented in the CLC GW. The differentially expressed transcripts (DETs) were extracted according to the following criteria: fold change ≥2, FDR *p*-corrected value ≤ 0.005, RPKM ≥ 3 in all samples. DETs were subjected to a hypergeometric test^38^ and *k*-mean clustered^39^ according to their change in expression level across the developmental stages.

### Validation of RNA-seq expression profiles using RT-qPCR

Total RNA was treated with DNase (Thermo Fischer Scientific) and cleaned using the RNeasy Micro Kit (Qiagen). The first cDNA strand was synthesized using Superscript II Reverse Transcriptase (Thermo Fischer Scientific) and oligo-dT primer, according to the manufacturer’s instructions. RT-qPCR was performed on a LightCycler Nano (Roche, Basel, Switzerland) with SYBRGreen (Thermo Fischer Scientific) as fluorescent probe, using 50 ng cDNA for each reaction. Cq values were used to calculate the relative transcript abundance following the described methods^51,52^. The primers used are listed in Supplementary Table S7.

### Comparative transcriptomics and transcriptional regulatory network

A list of *Arabidopsis* genes previously reported to be important regulators of root development was created by literature mining. The sequences of these genes, together with their interactions and first neighbours, were retrieved from the *Arabidopsis* gene transcriptional regulatory map^43^ (downloaded from http://atrm.cbi.pku.edu.cn/). The putative *P*. *pringlei* orthologues of these genes were identified by determining the best bidirectional hit (BBH) for each sequence when possible, or unidirectional hits when the BBH was unsuccessful. The contigs were considered as putative orthologues when the BLAST coverage was >40% and the E-value was <1 × 10^-10^, and when domains from the query sequence, verified by SuperFamily^53^, were present. The resulting transcriptional regulatory network (TRN) was visualized in Cytoscape^54^ (v3.2.1).

## Electronic supplementary material

Supplementary Information
