# Supplementary material for: Transcriptomics insights into the genetic regulation of root apical meristem exhaustion and determinate primary root growth in Pachycereus pringlei (Cactaceae)
Source: Sci Rep. 2018 Jun 4;8:8529. doi: 10.1038/s41598-018-26897-1 (PMC5986787; doi:10.1038/s41598-018-26897-1)
Supplement: Supplementary file 1 — Supplementary Information [file 41598_2018_26897_MOESM1_ESM.pdf]

Transcriptomics insights into the genetic regulation of root apical meristem exhaustion and determinate primary root growth in *Pachycereus pringlei* (Cactaceae)

Rodríguez-Alonso G.[1], Matvienko M.[2], López-Valle M. L.[1], Lázaro-Mixteco P. E.[1], Napsucialy-Mendivil S.[1], Dubrovsky J. G.[1] and Shishkova S.[1]*

[1] Departamento de Biología Molecular de Plantas, Instituto de Biotecnología, Universidad Nacional Autónoma de México, Cuernavaca, Mexico.
[2] Tecan Systems, 2450 Zanker Rd, San Jose, CA 95131, United States

* Corresponding author: sveta @ ibt.unam.mx

Supplementary Figure S1. B2GO annotation statistics for the *de novo* assembled transcriptome of the *Pachycereus pringlei* primary root apex. Note: the following stats are from a representative subset of the total contigs. Upper panel: top hit species during the B2GO annotation; lower panel: distribution of similarity values between *P. pringlei* translated contigs and the top hit sequences during B2GO annotation.

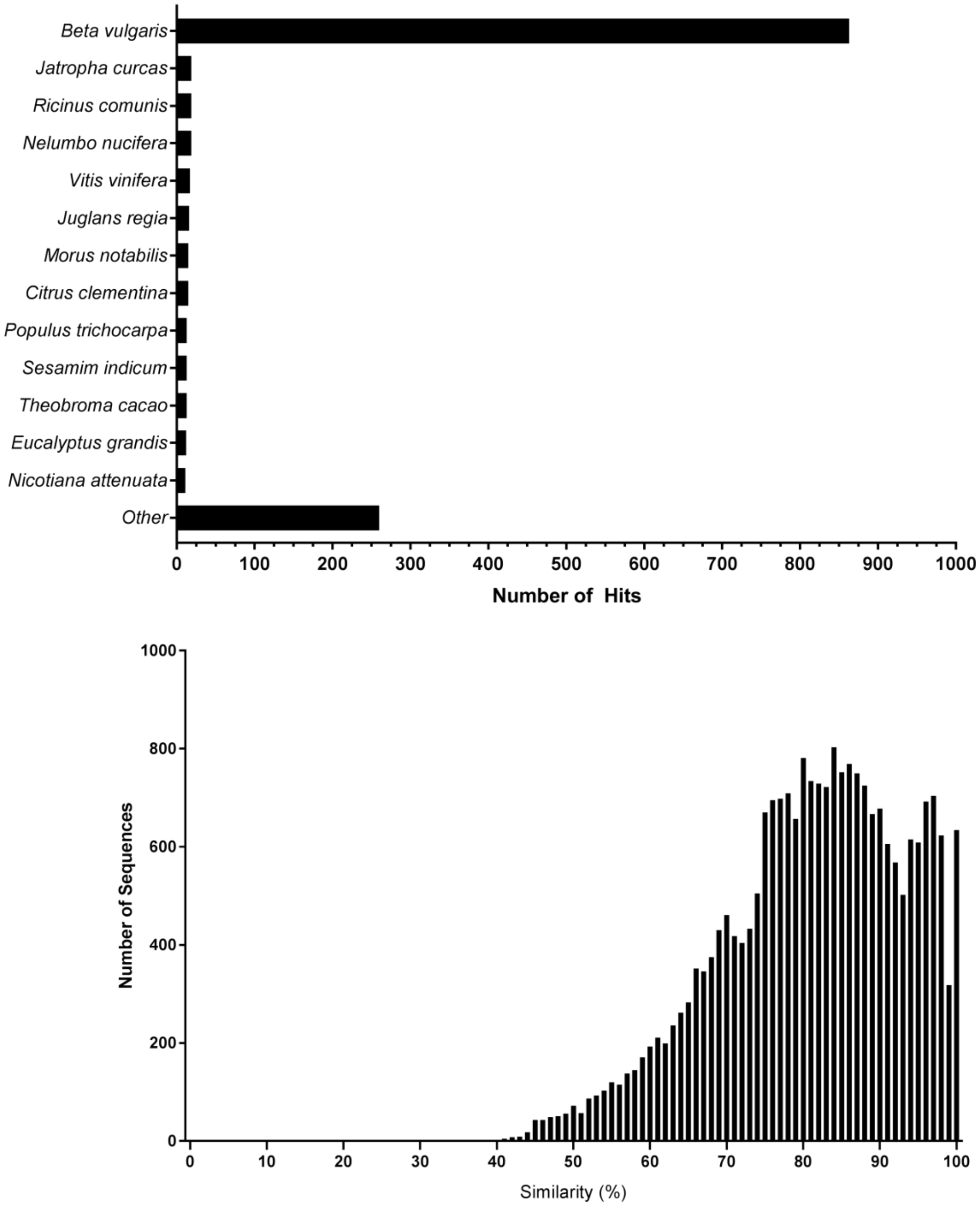

Supplementary Figure S2. The *Pachycereus pringlei* transcriptome is comprehensive. KEGG Automatic Annotation Server mapping of the *de novo* assembled primary root apex transcriptome of *P. pringlei* to some biological processes and pathways. The boxes in each process or pathway represent enzymes or structural proteins. Green boxes: at least one *P. pringlei* putative ortholog was identified in the transcriptome; white boxes: no *P. pringlei* putative ortholog was identified. Permission to use the schemes was kindly granted by Kanehisha Laboratories. Schemes were generated by the KAAS mapping tool (Moriya *et al.*, 2007, DOI:10.1093/nar/gkm321). Reference: Kanehisa M., Furumichi M., Tanabe M., Sato Y., Morishima K. KEGG: new perspectives on genomes, pathways, diseases and drugs. Nuc. Ac. Res. 45, D353-D361 (2017).

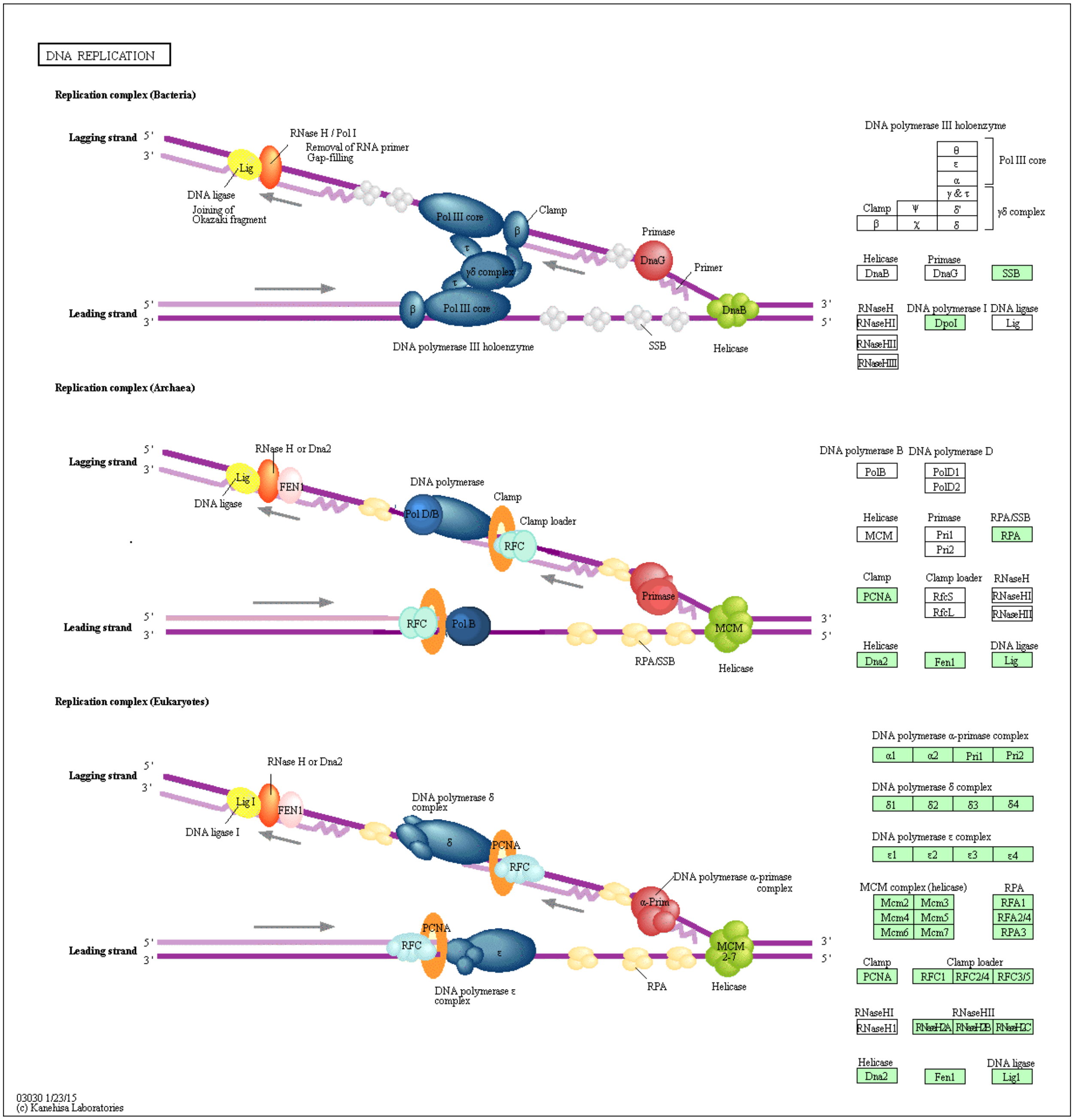

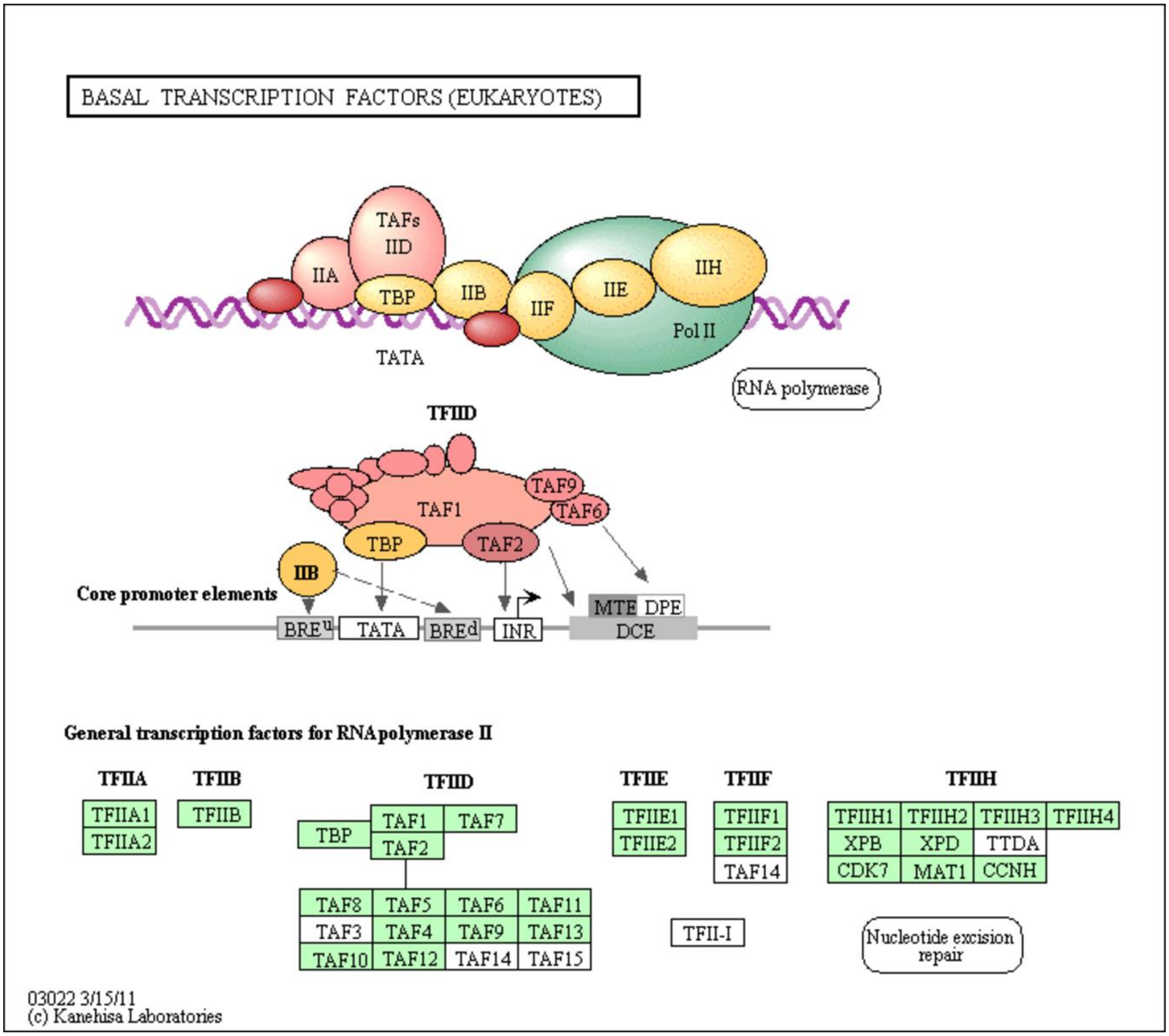
BASAL TRANSCRIPTION FACTORS (EUKARYOTES)
TAFs
IID
IIA
TBP
IIB
IIF
IIE
IIH
Pol II
TATA
RNA polymerase
TFIID
TAF9
TAF6
TAF1
TBP
TAF2
IIB
Core promoter elements
BRE u
TATA
BRE d
INR
MTE
DPE
DCE
General transcription factors for RNA polymerase II
TFIIA
TFIIA1
TFIIA2
TFIIB
TFIIB
TFIID
TBP
TAF1
TAF7
TAF2
TAF8
TAF5
TAF6
TAF11
TAF3
TAF4
TAF9
TAF13
TAF10
TAF12
TAF14
TAF15
TFIIE
TFIIE1
TFIIE2
TFIIF
TFIIF1
TFIIF2
TAF14
TFII-I
TFIIH
TFIIH1
TFIIH2
TFIIH3
TFIIH4
XPB
XPD
TTDA
CDK7
MAT1
CCNH
Nucleotide excision repair
03022 3/15/11
(c) Kanehisa Laboratories

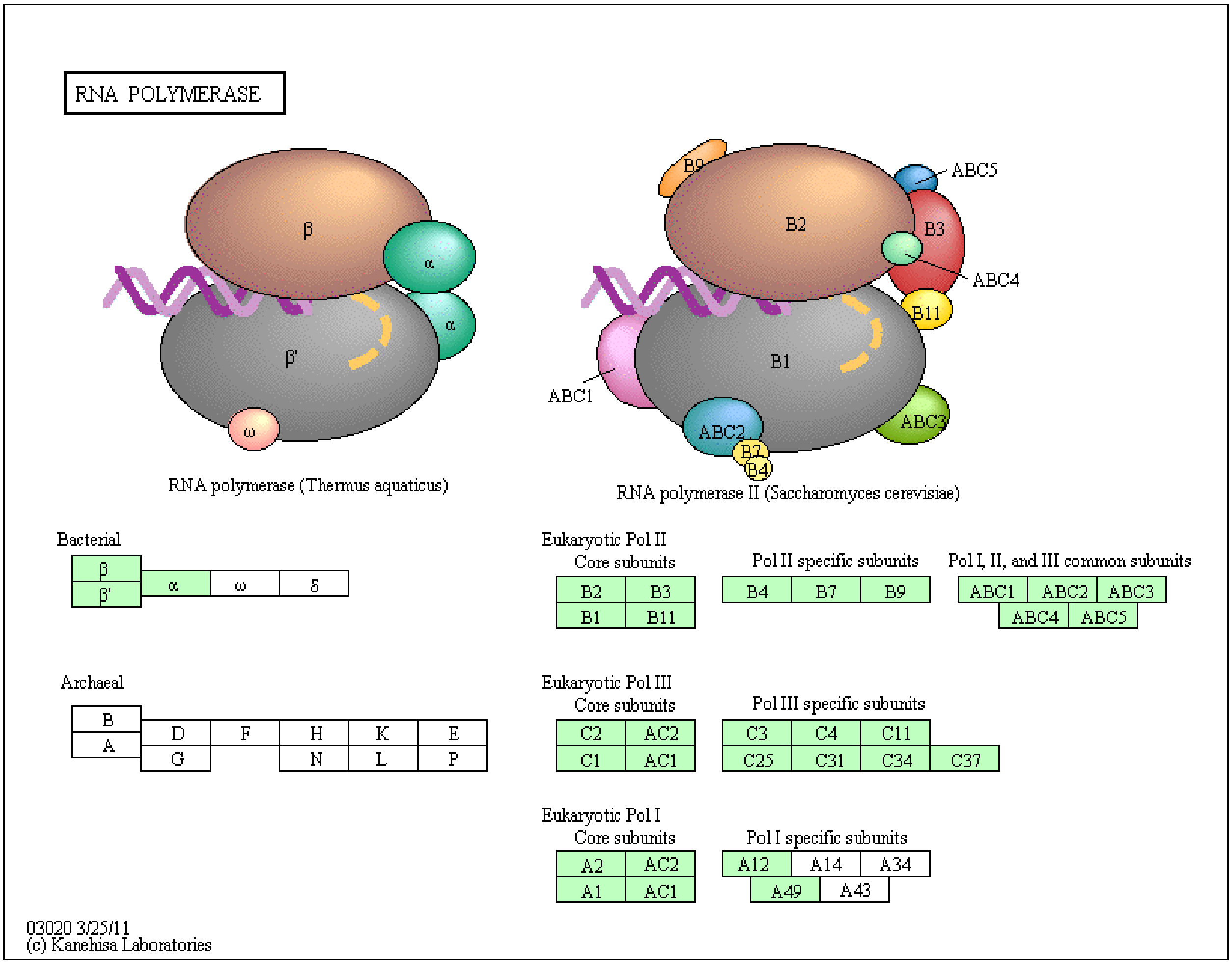
RNA POLYMERASE
β
α
α
β'
ω
RNA polymerase (Thermus aquaticus)
B9
B2
ABC5
B3
ABC4
B11
B1
ABC1
ABC2
ABC3
B7
B4
RNA polymerase II (Saccharomyces cerevisiae)
Bacterial
β
β'
α
ω
δ
Archaeal
B
A
D
G
F
H
N
K
L
E
P
Eukaryotic Pol II
Core subunits
B2
B3
B1
B11
Pol II specific subunits
B4
B7
B9
Pol I, II, and III common subunits
ABC1
ABC2
ABC3
ABC4
ABC5
Eukaryotic Pol III
Core subunits
C2
AC2
C1
AC1
Pol III specific subunits
C3
C4
C11
C25
C31
C34
C37
Eukaryotic Pol I
Core subunits
A2
AC2
A1
AC1
Pol I specific subunits
A12
A14
A34
A49
A43
03020 3/25/11
(c) Kanehisa Laboratories

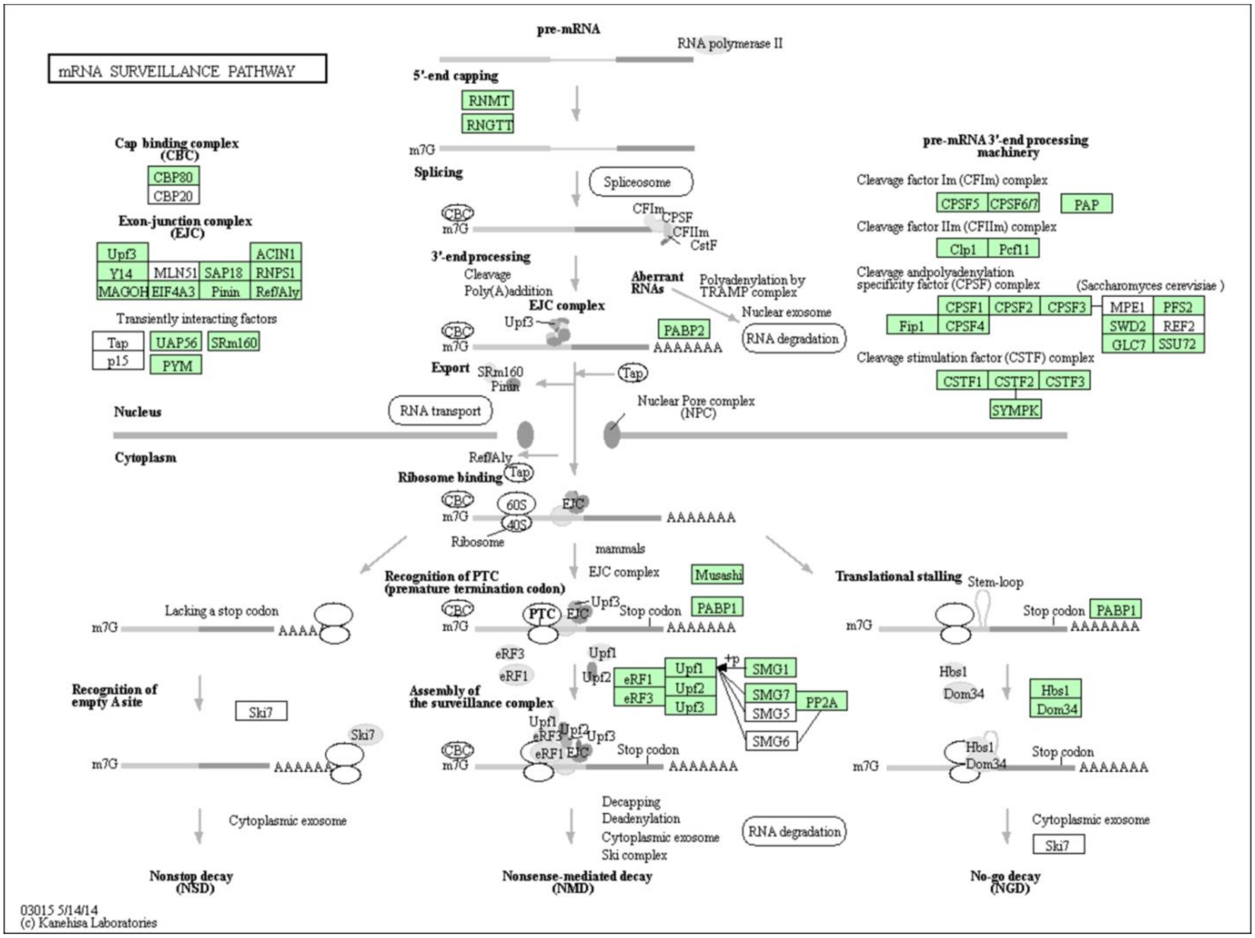

mRNA SURVEILLANCE PATHWAY
pre-mRNA
RNA polymerase II
5'-end capping
RNMT
RNGTT
Splicing
Spliceosome
3'-end processing
Cleavage
Poly(A)addition
EJC complex
Aberrant RNAs
Polyadenylation by TRAMP complex
Nuclear exosome
RNA degradation
Export
RNA transport
Nuclear Pore complex (NPC)
Nucleus
Cytoplasm
Ribosome binding
Cap binding complex (CBC)
CBP80
CBP20
Exon-junction complex (EJC)
Upf3
ACIN1
Y14
MLN51
SAP18
RNPS1
MAGOH
EIF4A3
Pinin
Ref/Aly
Transiently interacting factors
Tap
p15
UAP56
SRm160
PYM
pre-mRNA 3'-end processing machinery
Cleavage factor Im (CFIm) complex
CPSF5
CPSF6/7
PAP
Cleavage factor IIm (CFIIm) complex
Clp1
Pcf11
Cleavage andpolyadenylation specificity factor (CPSF) complex
(Saccharomyces cerevisiae)
CPSF1
CPSF2
CPSF3
MPE1
PFS2
Fip1
CPSF4
SWD2
REF2
GLC7
SSU72
Cleavage stimulation factor (CSTF) complex
CSTF1
CSTF2
CSTF3
SYMPK
PABP2
Recognition of PTC (premature termination codon)
Translational stalling
Lacking a stop codon
Stop codon
Stem-loop
Musashi
PABP1
Recognition of empty A site
Ski7
Assembly of the surveillance complex
eRF1
Upf1
Upf2
eRF3
SMG1
SMG7
PP2A
SMG5
SMG6
Hbs1
Dom34
Cytoplasmic exosome
Decapping
Deadenylation
Ski complex
Nonstop decay (NSD)
Nonsense-mediated decay (NMD)
No-go decay (NGD)
03015 5/14/14
(c) Kanehisa Laboratories

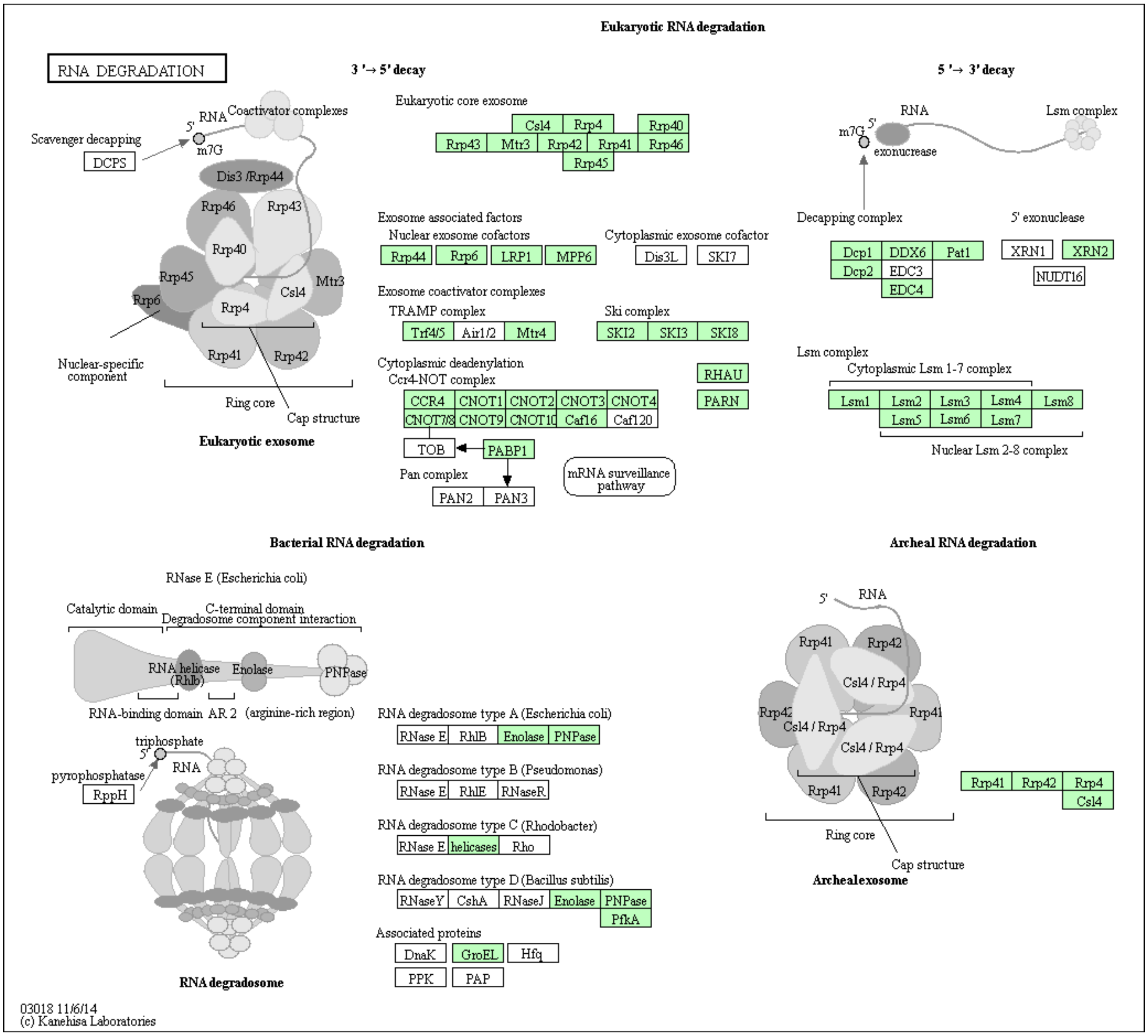
Eukaryotic RNA degradation
RNA DEGRADATION
3 '→ 5' decay
5 '→ 3' decay
Scavenger decapping
DCPS
RNA
Coactivator complexes
m7G
Dis3 /Rrp44
Rrp46
Rrp43
Rrp40
Rrp45
Rrp6
Mtr3
Csl4
Rrp4
Rrp41
Rrp42
Nuclear-specific component
Ring core
Cap structure
Eukaryotic exosome
Eukaryotic core exosome
Csl4 Rrp4 Rrp40
Rrp43 Mtr3 Rrp42 Rrp41 Rrp46
Rrp45
Exosome associated factors
Nuclear exosome cofactors
Rrp44 Rrp6 LRP1 MPP6
Cytoplasmic exosome cofactor
Dis3L SKI7
Exosome coactivator complexes
TRAMP complex
Trf4/5 Air1/2 Mtr4
Ski complex
SKI2 SKI3 SKI8
Cytoplasmic deadenylation
Ccr4-NOT complex
CCR4 CNOT1 CNOT2 CNOT3 CNOT4
CNOT7/8 CNOT9 CNOT10 Caf16 Caf120
RHAU
PARN
TOB
PABP1
Pan complex
PAN2 PAN3
mRNA surveillance pathway
RNA
Lsm complex
m7G
exonucrease
Decapping complex
Dcp1 DDX6 Pat1
Dcp2 EDC3
EDC4
5' exonuclease
XRN1 XRN2
NUDT16
Lsm complex
Cytoplasmic Lsm 1-7 complex
Lsm1 Lsm2 Lsm3 Lsm4 Lsm8
Lsm5 Lsm6 Lsm7
Nuclear Lsm 2-8 complex
Bacterial RNA degradation
RNase E (Escherichia coli)
Catalytic domain
C-terminal domain
Degradosome component interaction
RNA helicase (Rhlb)
Enolase
PNPase
RNA-binding domain AR 2 (arginine-rich region)
triphosphate
pyrophosphatase
RppH
RNA
RNA degradosome
RNA degradosome type A (Escherichia coli)
RNase E RhlB Enolase PNPase
RNA degradosome type B (Pseudomonas)
RNase E RhlE RNaseR
RNA degradosome type C (Rhodobacter)
RNase E helicases Rho
RNA degradosome type D (Bacillus subtilis)
RNaseY CshA RNaseJ Enolase PNPase
PfkA
Associated proteins
DnaK GroEL Hfq
PPK PAP
Archeal RNA degradation
RNA
Rrp41
Rrp42
Csl4 / Rrp4
Rrp42
Rrp41
Csl4 / Rrp4
Csl4 / Rrp4
Rrp41
Rrp42
Ring core
Cap structure
Archeal exosome
Rrp41 Rrp42 Rrp4
Csl4
03018 11/6/14
(c) Kanehisa Laboratories

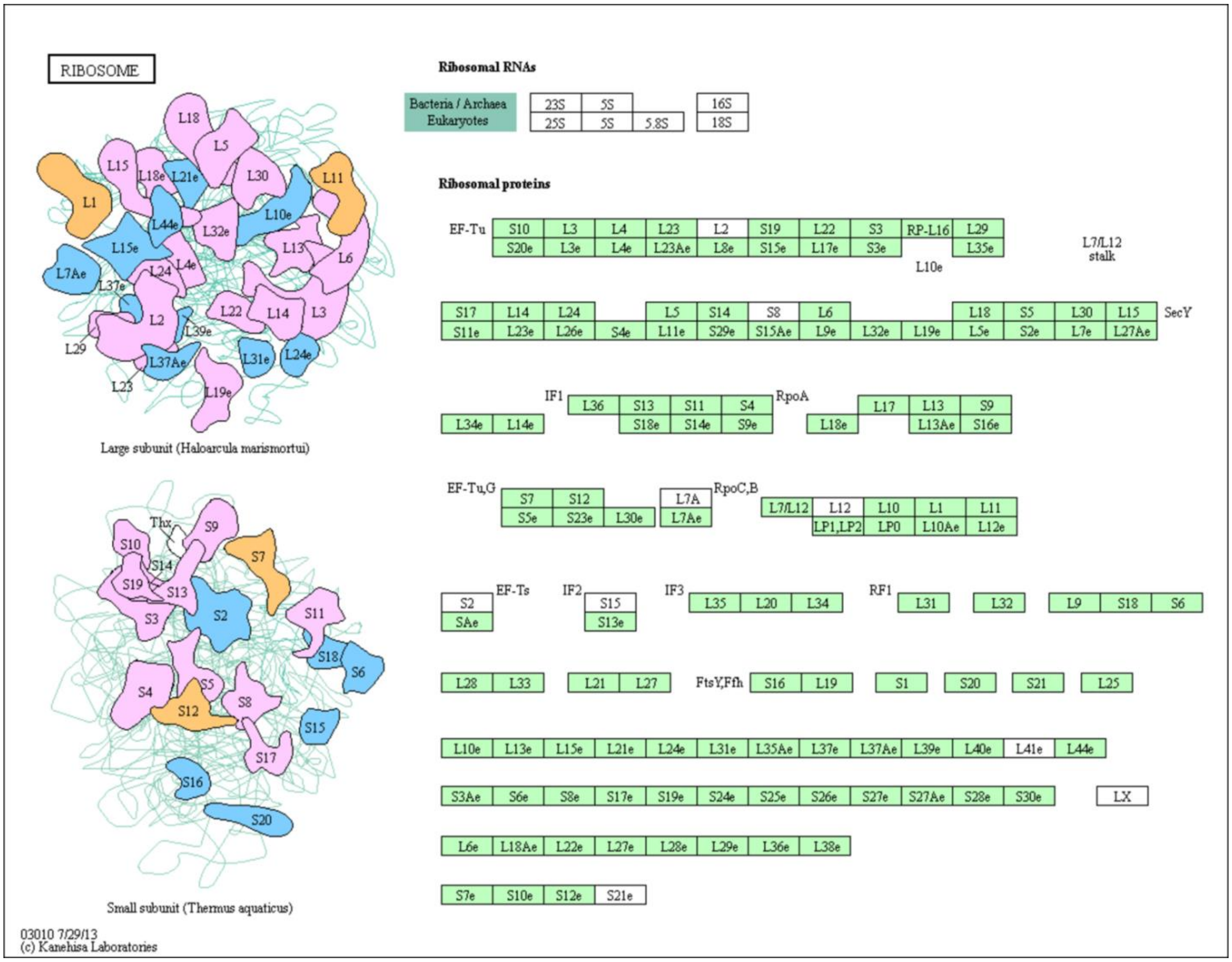

RIBOSOME
Ribosomal RNAs
Bacteria / Archaea
Eukaryotes
23S 5S 16S
25S 5S 5.8S 18S
Ribosomal proteins
EF-Tu
S10 L3 L4 L23 L2 S19 L22 S3 RP-L16 L29
S20e L3e L4e L23Ae L8e S15e L17e S3e L35e
L10e
L7/L12
stalk
S17 L14 L24 L5 S14 S8 L6 L18 S5 L30 L15 SecY
S11e L23e L26e S4e L11e S29e S15Ae L9e L32e L19e L5e S2e L7e L27Ae
IF1 RpoA
L36 S13 S11 S4 L17 L13 S9
L34e L14e S18e S14e S9e L18e L13Ae S16e
EF-Tu,G RpoC,B
S7 S12 L7A
S5e S23e L30e L7Ae
L7/L12 L12 L10 L1 L11
LP1,LP2 LP0 L10Ae L12e
EF-Ts IF2 IF3 RF1
S2 S15 L35 L20 L34 L31 L32 L9 S18 S6
SAe S13e
L28 L33 L21 L27 FtsY,Ffh S16 L19 S1 S20 S21 L25
L10e L13e L15e L21e L24e L31e L35Ae L37e L37Ae L39e L40e L41e L44e
S3Ae S6e S8e S17e S19e S24e S25e S26e S27e S27Ae S28e S30e LX
L6e L18Ae L22e L27e L28e L29e L36e L38e
S7e S10e S12e S21e
L18 L5 L15 L18e L21e L30 L11 L1 L10e L44e L32e L15e L13 L6 L7Ae L24 L4e L37e L2 L22 L14 L3 L39e L29 L37Ae L31e L24e L23 L19e
Large subunit (Haloarcula marismortui)
Thx S9 S10 S14 S7 S19 S13 S3 S2 S11 S18 S6 S4 S5 S12 S8 S15 S17 S16 S20
Small subunit (Thermus aquaticus)
03010 7/29/13
(c) Kanehisa Laboratories

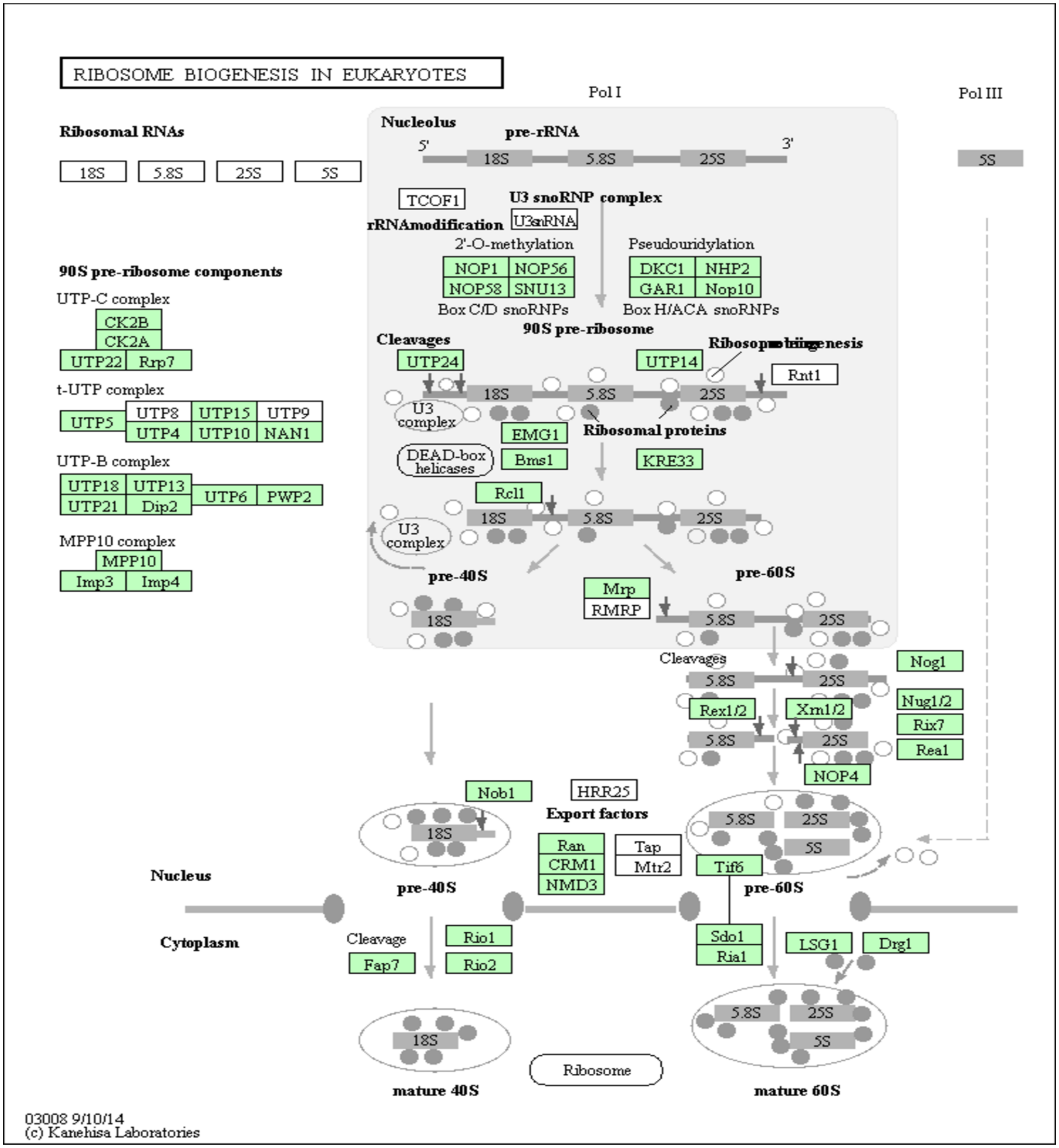
RIBOSOME BIOGENESIS IN EUKARYOTES
Pol I
Pol III
Nucleolus
pre-rRNA
5'
3'
18S
5.8S
25S
5S
Ribosomal RNAs
TCOF1
U3 snoRNP complex
U3snRNA
rRNAmodification
2'-O-methylation
Pseudouridylation
NOP1
NOP56
NOP58
SNU13
Box C/D snoRNPs
DKC1
NHP2
GAR1
Nop10
Box H/ACA snoRNPs
90S pre-ribosome components
UTP-C complex
CK2B
CK2A
UTP22
Rrp7
t-UTP complex
UTP5
UTP8
UTP15
UTP9
UTP4
UTP10
NAN1
UTP-B complex
UTP18
UTP13
UTP21
Dip2
UTP6
PWP2
MPP10 complex
MPP10
Imp3
Imp4
90S pre-ribosome
Cleavages
UTP24
UTP14
Rnt1
U3 complex
EMG1
Bms1
DEAD-box helicases
Ribosomal proteins
KRE33
Rcl1
pre-40S
pre-60S
Mrp
RMRP
Nog1
Nug1/2
Rix7
Rea1
Rex1/2
Xrn1/2
NOP4
Nob1
HRR25
Export factors
Ran
CRM1
NMD3
Tap
Mtr2
Tif6
Nucleus
Cytoplasm
Cleavage
Rio1
Fap7
Rio2
Sdo1
Ria1
LSG1
Drg1
Ribosome
mature 40S
mature 60S
03008 9/10/14
(c) Kanehisa Laboratories

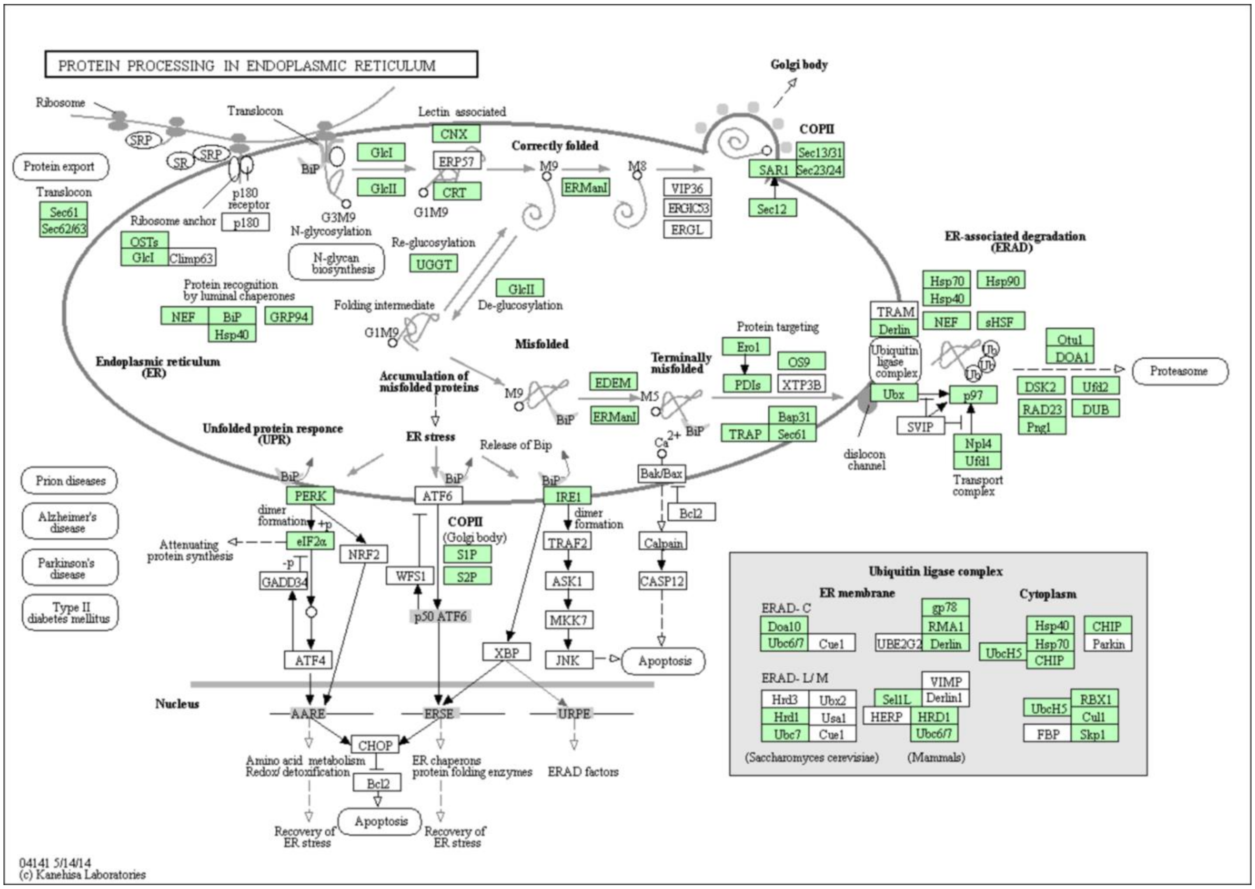

PROTEIN PROCESSING IN ENDOPLASMIC RETICULUM
Ribosome
Translocon
SRP
SR
Protein export
Translocon
Sec61
Sec62/63
p180 receptor
Ribosome anchor
p180
OSTs
GlcI
Climp63
BiP
G3M9
N-glycosylation
N-glycan biosynthesis
Lectin associated
CNX
GlcI
ERP57
GlcII
CRT
G1M9
Re-glucosylation
UGGT
Correctly folded
M9
ERManI
M8
VIP36
ERGIC53
ERGL
Golgi body
COPII
SAR1
Sec13/31
Sec23/24
Sec12
Protein recognition by luminal chaperones
NEF
BiP
Hsp40
GRP94
Folding intermediate
GlcII
De-glucosylation
G1M9
Endoplasmic reticulum (ER)
Accumulation of misfolded proteins
Misfolded
M9
EDEM
ERManI
M5
BiP
Terminally misfolded
Protein targeting
Ero1
PDIs
OS9
XTP3B
Bap31
TRAP
Sec61
Ca 2+
ER-associated degradation (ERAD)
TRAM
Derlin
Hsp70
Hsp40
Hsp90
NEF
sHSF
Ubiquitin ligase complex
Ub
Ubx
p97
SVIP
Npl4
Ufd1
Transport complex
dislocon channel
Otu1
DOA1
Proteasome
DSK2
Ufd2
RAD23
DUB
Png1
Unfolded protein responce (UPR)
ER stress
Release of Bip
BiP
PERK
ATF6
IRE1
Bak/Bax
Bcl2
dimer formation
+p
eIF2α
Attenuating protein synthesis
-p
GADD34
NRF2
WFS1
COPII (Golgi body)
S1P
S2P
p50 ATF6
dimer formation
TRAF2
ASK1
MKK7
JNK
Calpain
CASP12
Apoptosis
ATF4
XBP
Prion diseases
Alzheimer's disease
Parkinson's disease
Type II diabetes mellitus
Nucleus
AARE
ERSE
URPE
CHOP
Amino acid metabolism Redox/ detoxification
ER chaperons protein folding enzymes
ERAD factors
Bcl2
Apoptosis
Recovery of ER stress
Recovery of ER stress
Ubiquitin ligase complex
ER membrane
Cytoplasm
ERAD- C
Doa10
Ubc6/7
Cue1
gp78
RMA1
UBE2G2
Derlin
Hsp40
Hsp70
CHIP
CHIP
Parkin
UbcH5
ERAD- L/ M
Hrd3
Ubx2
Hrd1
Usa1
Ubc7
Cue1
VIMP
Sel1L
Derlin1
HERP
HRD1
Ubc6/7
UbcH5
RBX1
Cul1
FBP
Skp1
(Saccharomyces cerevisiae)
(Mammals)
04141 5/14/14
(c) Kanehisa Laboratories

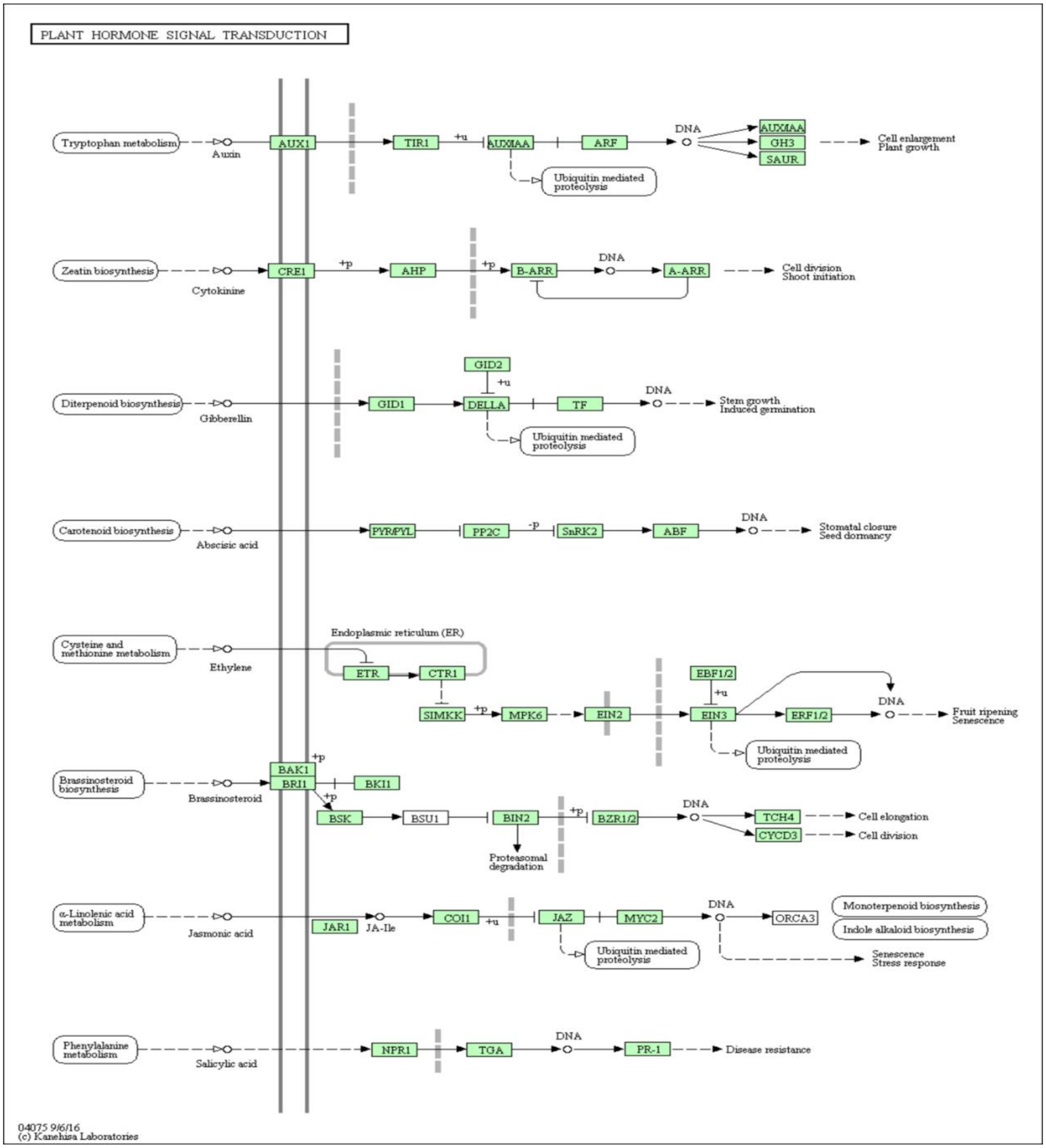
PLANT HORMONE SIGNAL TRANSDUCTION
Tryptophan metabolism
Auxin
AUX1
TIR1
+u
AUX/IAA
ARF
DNA
AUX/IAA
GH3
SAUR
Cell enlargement
Plant growth
Ubiquitin mediated proteolysis
Zeatin biosynthesis
Cytokinine
CRE1
+p
AHP
+p
B-ARR
DNA
A-ARR
Cell division
Shoot initiation
GID2
+u
Diterpenoid biosynthesis
Gibberellin
GID1
DELLA
TF
DNA
Stem growth
Induced germination
Ubiquitin mediated proteolysis
Carotenoid biosynthesis
Abscisic acid
PYR/PYL
PP2C
-p
SnRK2
ABF
DNA
Stomatal closure
Seed dormancy
Endoplasmic reticulum (ER)
Cysteine and methionine metabolism
Ethylene
ETR
CTR1
SIMKK
+p
MPK6
EIN2
EBF1/2
+u
EIN3
ERF1/2
DNA
Fruit ripening
Senescence
Ubiquitin mediated proteolysis
BAK1
+p
Brassinosteroid biosynthesis
Brassinosteroid
BRI1
BKI1
+p
BSK
BSU1
BIN2
+p
BZR1/2
DNA
TCH4
CYCD3
Cell elongation
Cell division
Proteasomal degradation
α-Linolenic acid metabolism
Jasmonic acid
JAR1
JA-Ile
COI1
+u
JAZ
MYC2
DNA
ORCA3
Monoterpenoid biosynthesis
Indole alkaloid biosynthesis
Ubiquitin mediated proteolysis
Senescence
Stress response
Phenylalanine metabolism
Salicylic acid
NPR1
TGA
DNA
PR-1
Disease resistance
04075 9/6/16
(c) Kanehisa Laboratories

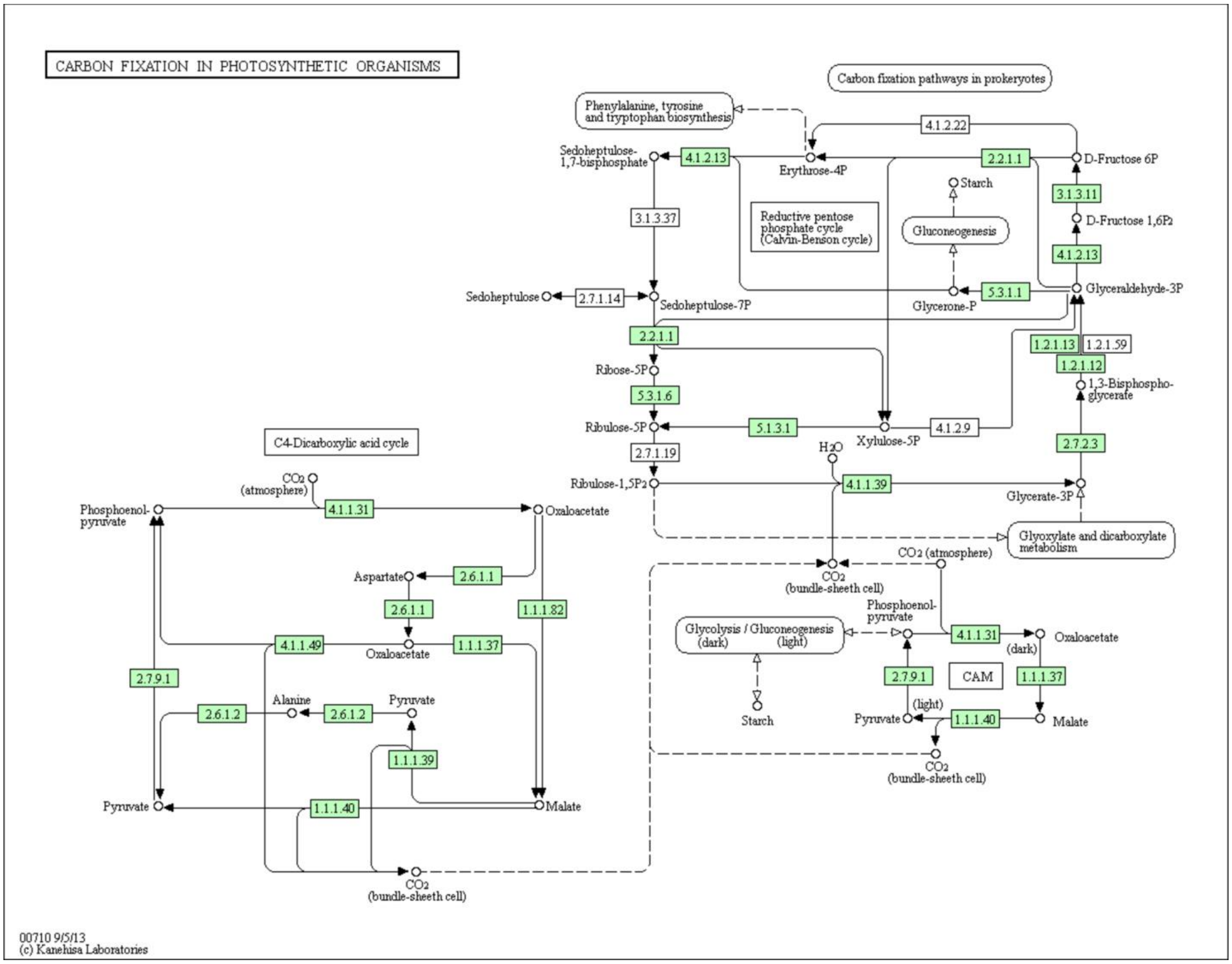

CARBON FIXATION IN PHOTOSYNTHETIC ORGANISMS
Carbon fixation pathways in prokeryotes
Phenylalanine, tyrosine and tryptophan biosynthesis
4.1.2.22
Sedoheptulose-1,7-bisphosphate
4.1.2.13
Erythrose-4P
2.2.1.1
D-Fructose 6P
Starch
3.1.3.11
3.1.3.37
Reductive pentose phosphate cycle (Calvin-Benson cycle)
Gluconeogenesis
D-Fructose 1,6P2
4.1.2.13
Sedoheptulose
2.7.1.14
Sedoheptulose-7P
Glycerone-P
5.3.1.1
Glyceraldehyde-3P
2.2.1.1
1.2.1.13
1.2.1.59
1.2.1.12
Ribose-5P
1,3-Bisphospho-glycerate
5.3.1.6
Ribulose-5P
5.1.3.1
Xylulose-5P
4.1.2.9
C4-Dicarboxylic acid cycle
2.7.1.19
H2O
2.7.2.3
CO2 (atmosphere)
Ribulose-1,5P2
4.1.1.39
Glycerate-3P
Phosphoenol-pyruvate
4.1.1.31
Oxaloacetate
Glyoxylate and dicarboxylate metabolism
CO2 (bundle-sheeth cell)
CO2 (atmosphere)
Aspartate
2.6.1.1
2.6.1.1
1.1.1.82
Phosphoenol-pyruvate
Glycolysis / Gluconeogenesis (dark) (light)
4.1.1.31
Oxaloacetate
4.1.1.49
Oxaloacetate
1.1.1.37
(dark)
2.7.9.1
2.7.9.1
CAM
1.1.1.37
Alanine
Pyruvate
Starch
2.6.1.2
2.6.1.2
(light)
Pyruvate
1.1.1.40
Malate
1.1.1.39
CO2 (bundle-sheeth cell)
Pyruvate
1.1.1.40
Malate
CO2 (bundle-sheeth cell)
00710 9/5/13
(c) Kanehisa Laboratories

Supplementary Figure S3. Scree plot of the principal components for the *Pachycereus pringlei* samples used in this study. The cumulative variance of the two first principal components is 96.2%.

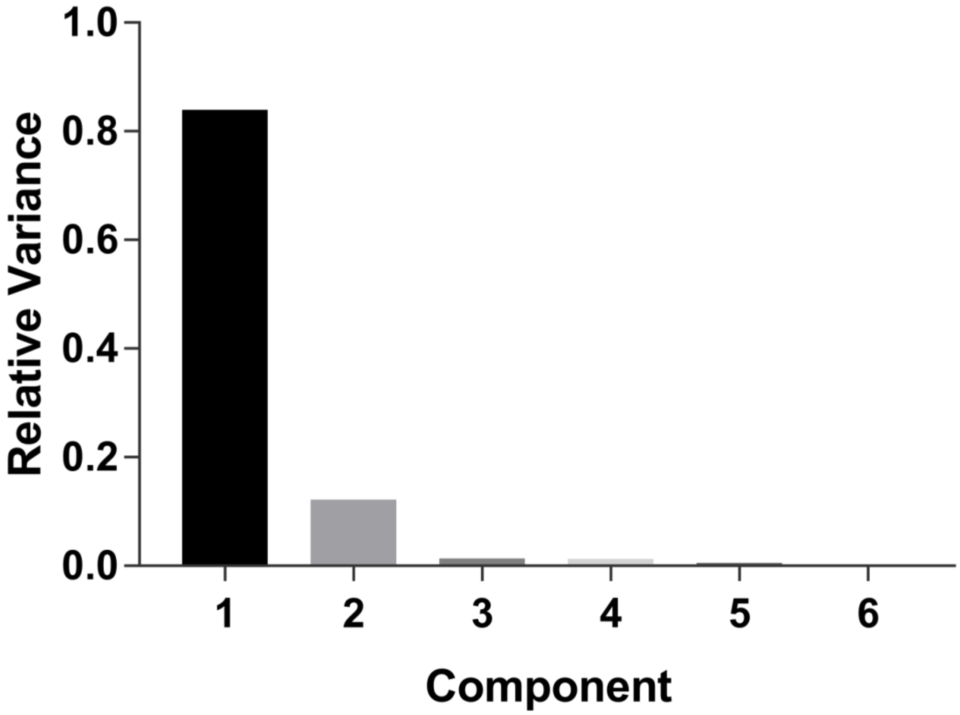

Supplementary Figure S4. Transcriptional profiles identified by *k*-mean clustering of the differentially expressed transcripts (DETs). Each plot depicts the normalized and centered expression level of the DETs at the three developmental stages of the *Pachycereus pringlei* primary root apex: IN, initial; IT, intermediate; TR, terminal.

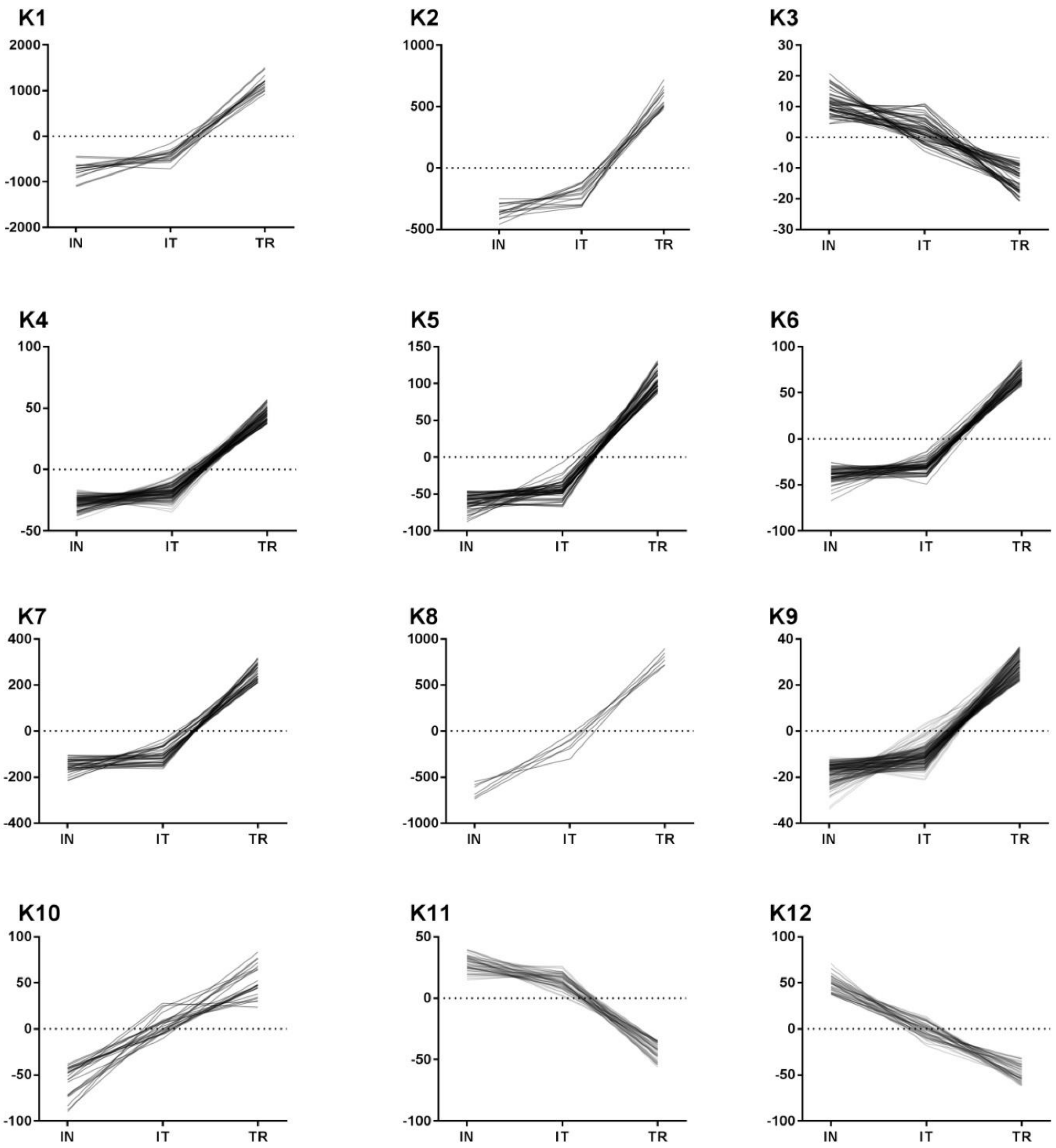

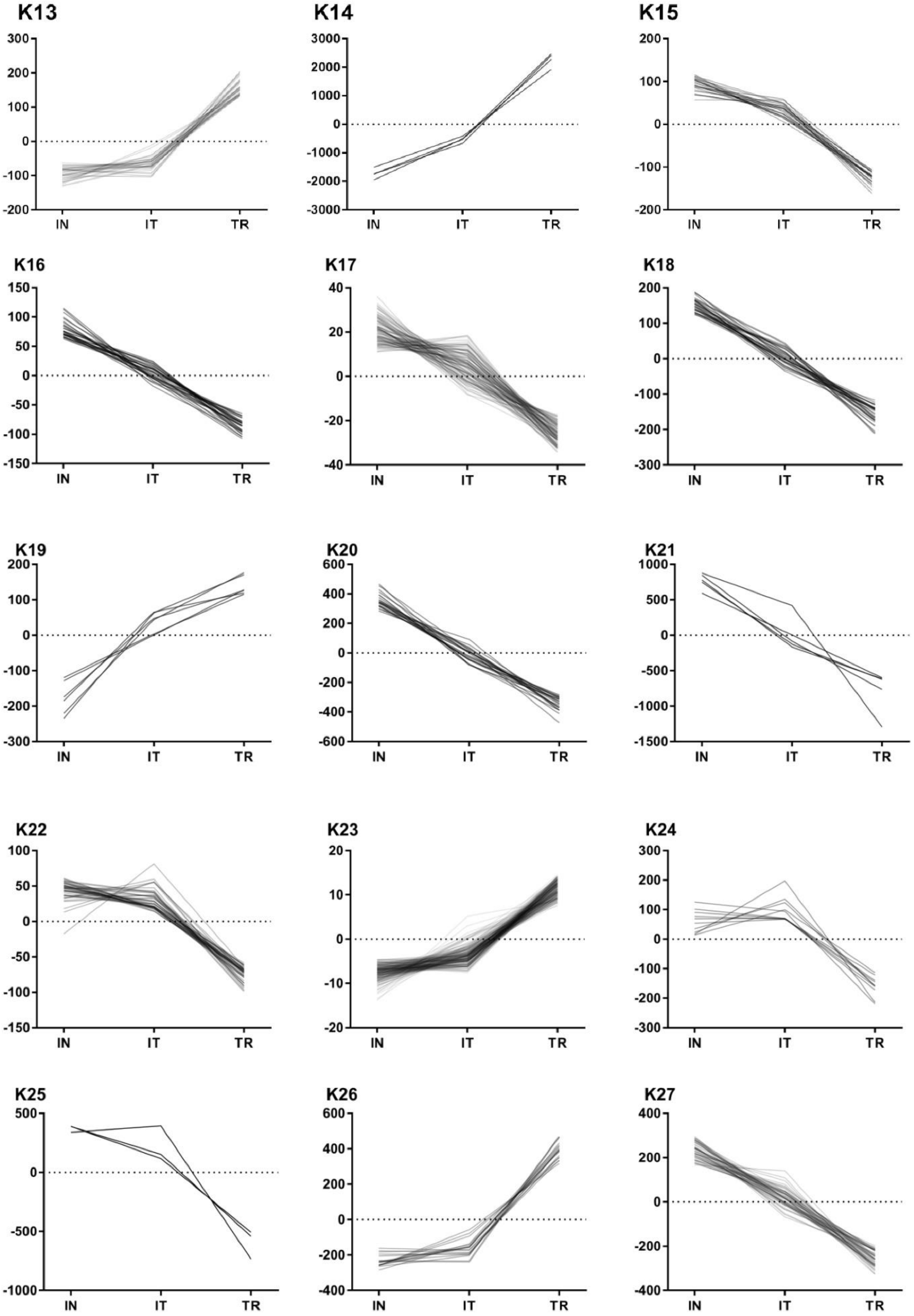
K13
K14
K15
K16
K17
K18
K19
K20
K21
K22
K23
K24
K25
K26
K27
IN
IT
TR

Supplementary Figure S5. Summary plot of the BlastN of the *Lophophora williamsii* shoot+root transcriptome against the *de novo* assembled transcriptome of the primary root apex of *Pachycereus pringlei*.

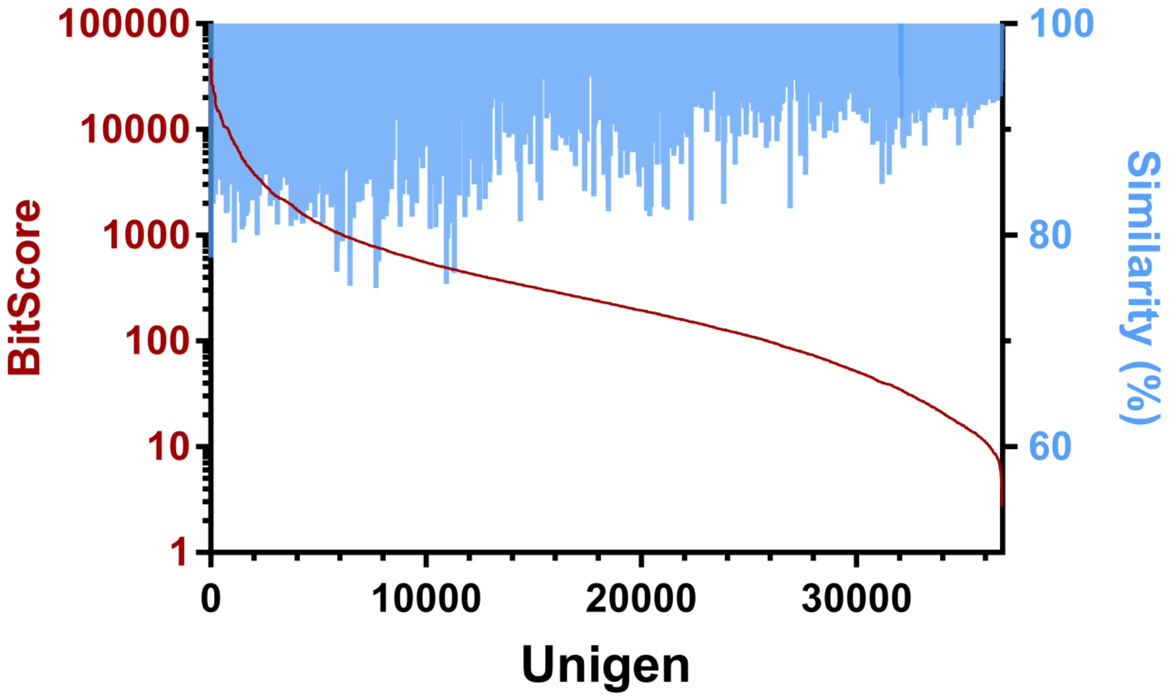

Supplementary Figure S6. Length distribution of the putative open reading frames identified in the un-annotated *Pachycereus pringlei* differentially expressed transcripts.

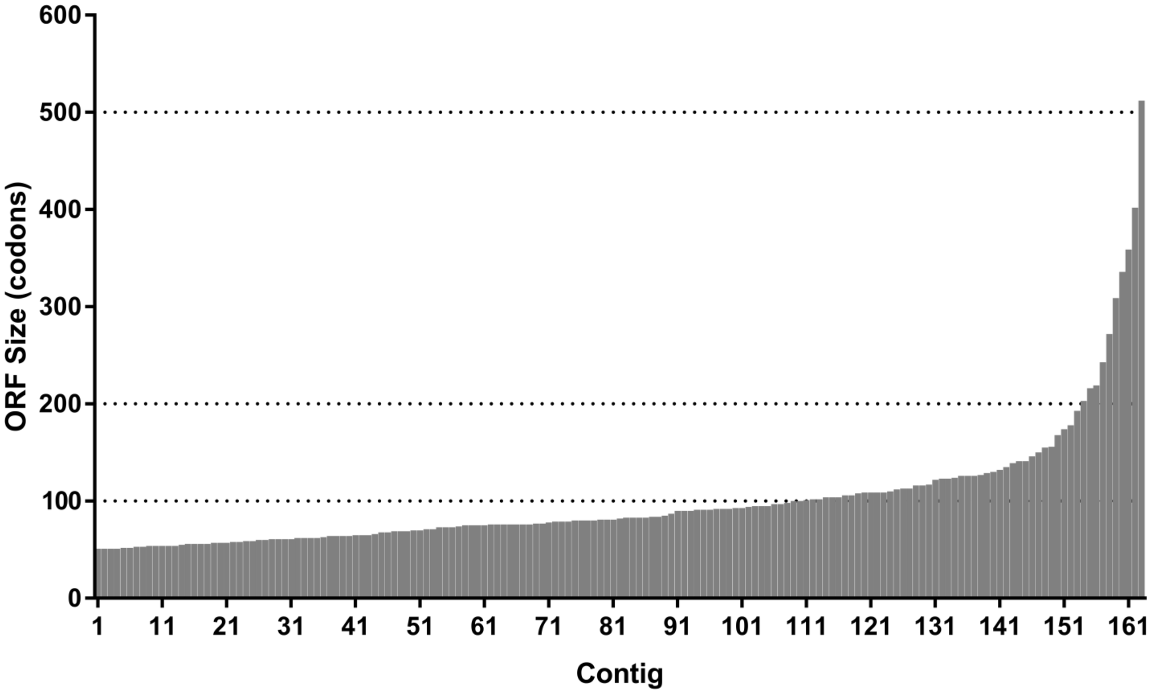

Supplementary Figure S7. Expression levels (RPKM values) of the *Pachycereus pringlei* candidate reference genes in the primary root apex at the initial and terminal developmental stages. Red diamonds correspond to the selected reference genes for this study.

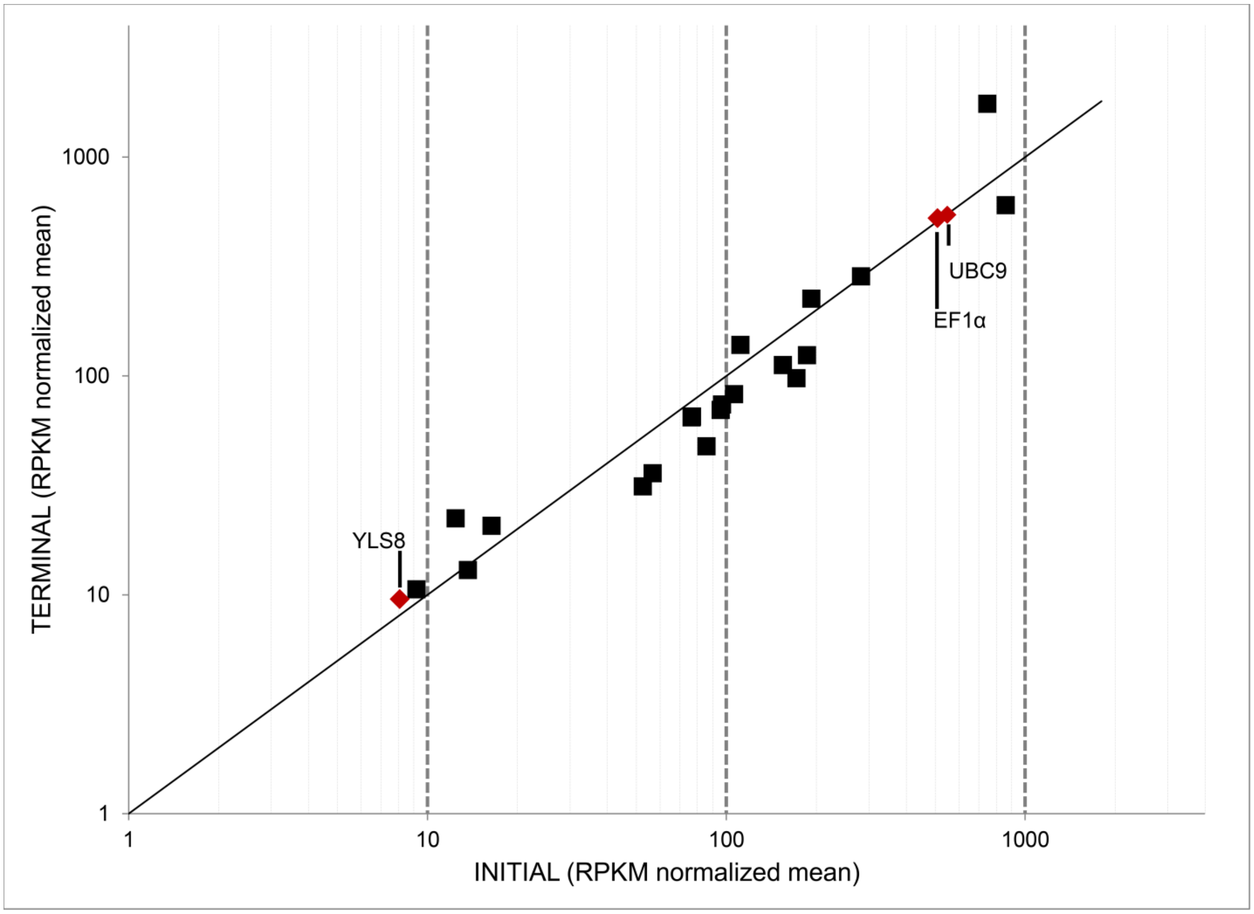

Supplementary Figure S8. Average RPKM values of the *Pachycereus pringlei* transcripts quantified by RT-qPCR. * the trend for RPKM values and transcript abundance evaluated by qRT-PCR between initial and terminal development stages was different for this transcript.

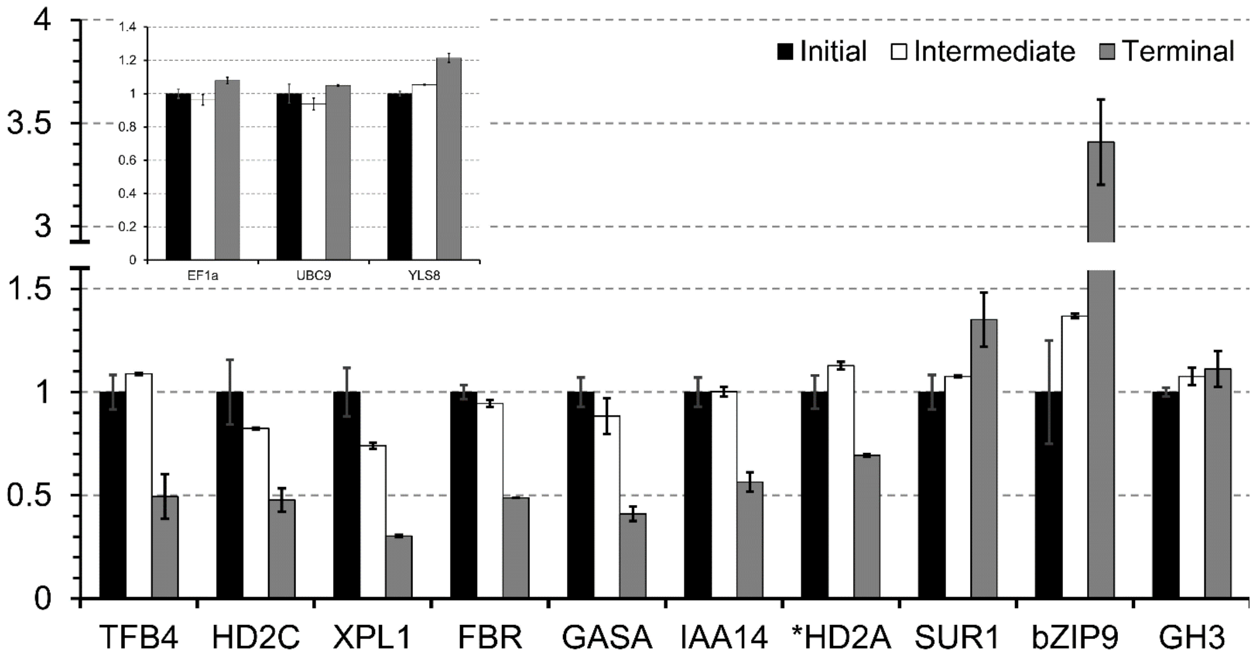

Supplementary Figure S9. Inferred transcriptional regulatory network for the *Pachycereus pringlei* primary root apex. The node size is proportional to the average expression level (log2(RPKM)) of the transcript in the *P. pringlei* transcriptome.

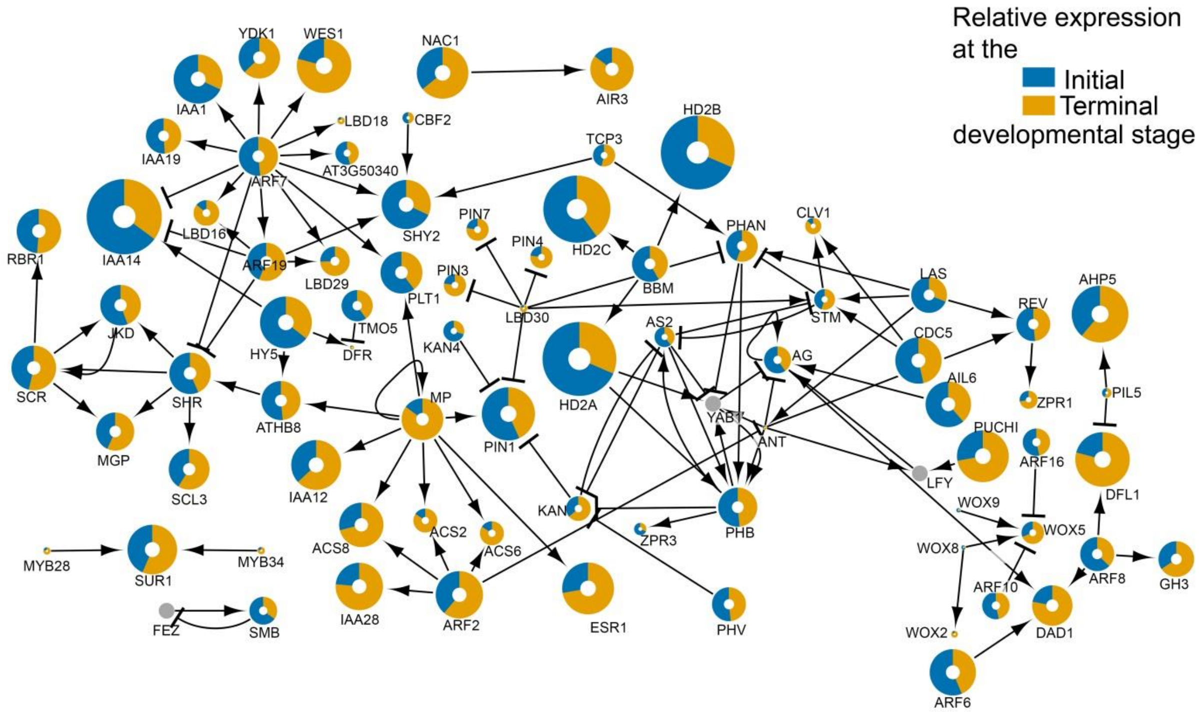

Supplementary Table S1. Number of differentially expressed genes for developmental zones of the primary root of six angiosperm species.

	EDGE MZ vs EZ		EDGE MZ vs DZ	
Species	MZ	EZ	MZ	DZ
Arabidopsis thaliana	2,683	4,432	3,734	8,947
Cucumis sativus	601	380	1,341	301
Glycine max	1,357	4,159	3,866	7,685
Oryza sativa	772	1,655	3,084	7,743
Solanum lycopersicum	2,905	3,542	4,125	5,661
Zea mays	2,018	3,285	8,012	8,563

* With the exception of *Cucumis sativus*, the number of up-regulated genes at the meristematic zone is lower in comparison to the up-regulated genes at the elongation or differentiation zone. This exception may arise from the fact that in *C. sativus*, unlike all the other species included in this table, new lateral root primordia are developed within meristematic zone (moreover, *C. sativus* develops the first lateral root primordia since the embryo development) (Dubrovsky & Rost, 2003).

Row data taken from Huang & Schiefelbein, 2015.

Dubrovsky J.G. & Rost T.L. 2003. Lateral root initiation. Encyclopedia of Applied Plant Sciences. Ed. by Thomas B., Murphy D.J., and Murray B.G. Academic Press (ISBN:0122270509) pp: 1101-1107.

Supplementary Table S2. Comparison of *Lophophora williamsii* and *Pachycereus pringlei de novo* assembled transcriptomes.

		*Lophophora**	*Pachycereus*
Sequencing	Platform	IonTorrent / 454	Illumina HiSeq
	Reads:	Single End	Paired End
	Total Reads	$6x10^{6}$	$280x10^{6}$
	Read Size (nt)	50-150	100 (x2)
Assembly	Min. contig Length (nt)	200	400
	Max. contig Length (nt)	4,170	14,583
	Av. contig Length (nt)	572	1,081
	Median contig Length (nt)	454	728
	Total contigs	40,436	49,045

* Data taken from Ibarra-Laclette *et al.*, 2015.

Supplementary Table S3. BlastN statistics from the comparison of *Lophophora williamsii* shoot+root transcriptome and *Pachycereus pringlei* root tip transcriptome.

	Minimum	Maximum	Average	Median
Identity	75	100	99.26	100
Bit Score	20.02	47,628.06	1,054.18	261.38
HSP*	1	270	21.6	21
E-value	0	9.75	0.02	0

* HSP: High-scoring significant pair.

Supplementary Table S4. *Pachycereus pringlei* putative orthologs of the *Arabidopsis* superior reference genes (Czechowski *et al*., 2005). The less variable contigs are included in the box. The asterisks indicate the reference genes used in this study.

Arabidopsis name	*P. pringlei* Contig	INITIAL		INTERMEDIATE		TERMINAL	
		RPKM 1	RPKM 2	RPKM 1	RPKM 2	RPKM 1	RPKM 2
AcylCoA BP	03269	73.21	58.6	66.48	66.63	38.02	38.08
PP2A	05248	142.42	121.42	123.2	123.98	77.23	78.74
AcylCoA BP	02079	43.93	36.76	39.08	40.8	24.55	25.43
CLA	09503	44.57	42.57	45.06	40.6	26.73	30.67
PP2A	09902	149.25	136.93	142.45	131.55	99.33	99.38
E2Fα	05001	717.61	604.83	684.04	727.78	545	415.74
PP2A	08913	112.74	124.83	112.59	100.72	88.43	90.68
CLA	12003	76.83	70.33	70.34	74.13	51.9	55.87
PP2A	07793	73.44	75.34	66.15	55.63	53.3	65.22
PP2A	04543	84.65	78.54	76.9	77.21	61.8	70.07
UBC	22624	59.02	58.65	56.54	54.67	48.15	55.25
KOR1	03527	58.84	59.27	64.18	72.66	54.75	49.39
KOR1	22902	10.52	10.46	14.94	17.09	10.13	10.62
**UBC**	**24691**	**414.88**	**427.47**	**396.49**	**377.1**	**402.53**	**470.79**
**UBC9***	**15035**	**229.12**	**204.45**	**210.96**	**195.78**	**226.4**	**228.57**
**E1Fα***	**06759**	**401.27**	**379.59**	**363.6**	**388.22**	**428.72**	**413.51**
GalaOx	***03600***	***7.44***	***6.67***	***8.34***	***8.38***	***9.1***	***7.8***
**YLS8***	**17282**	**145.98**	**150.19**	**156.32**	**155.68**	**183.81**	**175.92**
AGL31	***07058***	***5.9***	***6.49***	***5.29***	***7.16***	***7.85***	***7.45***
UBC	17222	80.47	90.87	89.78	90.51	100.3	121.37
PTB	48438	12.67	12.51	16.71	20.22	16.55	16.46
YLS8	30952	9.48	9.62	7.07	7.67	19.24	16.42
UBC	35678	525.63	622.83	655.12	672.13	1,546.79	1,249.75

Supplementary Table S5. Interaction of the *Arabidopsis* nodes used for the *Pachycereus pringlei* transcriptional regulatory network.

Node A	Interaction type	Node B
AT1G30330	Activation	AT2G44810
AT1G30490	Repression	AT5G16560
AT5G16560	Repression	AT1G65620
AT5G16560	Repression	AT1G73590
AT1G09770	Activation	AT1G62360
AT1G09770	Activation	AT1G75820
AT1G62360	Repression	AT2G37630
AT1G62360	Repression	AT1G65620
AT1G62360	Activation	AT1G75820
AT2G45190	Repression	AT4G18960
AT2G45190	Activation	AT2G34710
AT4G18960	Activation	AT2G44810
AT4G18960	Activation	AT4G18960
AT2G34710	Activation	AT1G65620
AT2G34710	Repression	AT5G16560
AT2G34710	Activation	AT2G45190
AT2G34710	Activation	AT3G52770
AT2G37630	Activation	AT2G34710
AT2G37630	Repression	AT2G45190
AT1G65620	Repression	AT2G45190
AT1G65620	Activation	AT2G34710
AT1G65620	Repression	AT1G62360
AT1G65620	Repression	AT5G16560
AT4G37750	Activation	AT5G61850
AT4G37750	Activation	AT5G60690
AT4G37750	Repression	AT4G18960
AT4G37750	Activation	AT2G45190
AT4G37750	Activation	AT2G34710
AT5G42630	Repression	AT1G73590
AT5G45980	Activation	AT5G59340
AT5G45980	Activation	AT3G11260
AT5G62000	Activation	AT1G01480
AT5G62000	Activation	AT4G11280
AT5G62000	Repression	AT4G37750
AT5G62000	Activation	AT4G37770
AT5G62000	Activation	AT5G25890
AT2G28350	Repression	AT3G11260
AT5G61850	Activation	AT4G18960

AT5G60690	Activation	AT2G45450
AT3G44750	Activation	AT2G34710
AT3G44750	Activation	AT2G45190
AT1G55580	Activation	AT1G62360
AT1G55580	Activation	AT2G37630
AT1G55580	Activation	AT5G60690
AT1G55580	Activation	AT4G37750
AT1G79580	Repression	AT1G26870
AT1G26870	Activation	AT1G79580
AT1G56010	Activation	AT2G04160
AT3G25710	Repression	AT5G42800
AT4G30080	Repression	AT3G11260
AT4G25470	Activation	AT1G04240
AT4G37650	Activation	AT5G03150
AT4G37650	Activation	AT3G54220
AT4G37650	Activation	AT1G03840
AT4G37650	Activation	AT1G50420
AT5G03150	Activation	AT3G54220
AT3G54220	Activation	AT1G03840
AT3G54220	Activation	AT3G12280
AT3G54220	Activation	AT5G03150
AT5G11260	Activation	AT4G32880
AT5G11260	Activation	AT4G14550
AT5G11260	Activation	AT5G42800
AT4G32880	Activation	AT4G37650
AT5G10510	Activation	AT4G18960
AT1G19850	Activation	AT3G20840
AT1G19850	Activation	AT4G11280
AT1G19850	Activation	AT4G32880
AT1G19850	Activation	AT4G37770
AT1G19850	Activation	AT1G04550
AT1G19850	Activation	AT1G12980
AT1G19850	Activation	AT1G19850
AT1G19850	Activation	AT1G73590
AT1G19850	Activation	AT1G01480
AT5G61420	Activation	AT2G20610
AT2G33880	Activation	AT3G11260
AT1G53230	Activation	AT2G37630
AT1G53230	Activation	AT1G04240
AT1G19220	Repression	AT4G14550
AT1G19220	Repression	AT4G37650
AT1G19220	Activation	AT2G42430
AT1G19220	Activation	AT3G58190

AT1G19220	Activation	AT1G04240
AT2G20180	Activation	AT1G03430
AT2G20180	Repression	AT5G54510
AT5G20730	Activation	AT3G20840
AT5G20730	Activation	AT3G15540
AT5G20730	Activation	AT3G58190
AT5G20730	Activation	AT3G50340
AT5G20730	Activation	AT1G19220
AT5G20730	Activation	AT1G04240
AT5G20730	Activation	AT2G45420
AT5G20730	Activation	AT2G42430
AT5G20730	Repression	AT4G37650
AT5G20730	Activation	AT4G14560
AT5G20730	Repression	AT4G14550
AT5G20730	Activation	AT4G37390
AT5G20730	Activation	AT4G27260
AT5G17430	Activation	AT3G44750
AT5G17430	Activation	AT5G03740
AT5G17430	Activation	AT5G22650
AT5G18560	Activation	AT5G61850
AT5G37020	Activation	AT1G28130
AT5G37020	Activation	AT2G44810
AT5G37020	Activation	AT5G54510
AT5G60890	Activation	AT2G20610
AT4G00220	Repression	AT2G37630
AT4G00220	Repression	AT2G01420
AT4G00220	Repression	AT1G73590
AT4G00220	Repression	AT1G70940
AT4G00220	Activation	AT1G62360
AT4G00220	Repression	AT1G23080

Supplementary Table S6. Properties of the nodes included in the *Pachycereus pringlei* transcriptional regulatory network of the primary root apex. NSH=No significant hit; NHF=No hit found; Y= Yes; N=No; Interm.=Intermediate.

Node	*Arabidopsis* name	*P. pringlei* contig	*P. pringlei* annotation	E-value	BBH	RPKM Initial	RPKM Interm.	RPKM Term.
AT1G30330	ARF6	0001038	auxin response factor 6-like	0	Y	24.12	27.34	18.65
AT2G44810	DAD1	0005103	phospholipase a1-chloroplastic-like	2.52E-75	N	7.27	9.93	25.76
AT1G30490	PHV	0001618	homeobox-leucine zipper protein revoluta	0	Y	7.93	9.77	7.39
AT5G16560	KAN	0005141	probable transcription factor kan2-like	4.24E-47	N	2.62	4.36	4.6
AT1G09770	CDC5	0000721	cell division cycle 5-like isoform x1	0	Y	23.08	26.9	20.04
AT1G62360	STM	0009327	homeobox protein knotted-1-like 6	3.66E-72	N	2.52	2.83	3.05
AT1G75820	CLV1	0001781	receptor protein kinase clavata1-like	0	Y	0.6	1.5	3.68
AT2G45190	YAB1	NSH	NSH	3.51	NSH	0.001	0.001	0.001
AT4G18960	AG	0032278	mads-box transcription factor 27-like isoform x1	2.62E-44	N	6.33	3.48	4.25
AT2G34710	PHB	0000849	homeobox-leucine zipper family protein	0	Y	12.14	14.87	11.42
AT2G37630	PHAN	0008775	transcription factor as1	1.94E-118	Y	5.7	8.01	7.17
AT1G65620	AS2	0027369	lob domain-containing protein 6	1.71E-62	Y	2.89	3.22	1.86
AT1G73590	PIN1	0002874	auxin efflux carrier component 1-like	0	Y	44.07	46	33.59
AT3G52770	ZPR3	0030394	PREDICTED: uncharacterized protein LOC101514572	1.15E-08	Y	1.74	1.66	0.78
AT4G37750	ANT	0014765	ap2-like ethylene-responsive transcription factor ant-like	1.99E-113	Y	0.53	0.34	1.28
AT5G42630	KAN4	0013106	probable transcription factor kan2-like	6.47E-35	N	3.3	3.75	1.42
AT5G45980	WOX8	0045416	wuschel-related homeobox 11-like	7.83E-16	N	1.15	0.48	0.54
AT5G59340	WOX2	0033393	wuschel-related homeobox 4	1.52E-21	N	0.33	0.39	1.91
AT3G11260	WOX5	0019483	wuschel-related homeobox 5-like	2.28E-51	Y	1.77	3.98	3.94
AT5G62000	ARF2	0001054	auxin response factor 2	0	N	19.42	26.39	30.43
AT1G01480	ACS2	0006764	1-aminocyclopropane-1-carboxylate synthase-	0	Y	1.28	2.89	6.82

			like					
AT4G11280	ACS6	0006764	1-aminocyclopropane-1-carboxylate synthase-like	0	Y	1.28	2.89	6.82
AT4G37770	ACS8	0007505	1-aminocyclopropane-1-carboxylate synthase-like	0	Y	9.92	25.33	24.34
AT5G25890	IAA28	0005636	auxin-responsive protein iaa26-like	5.59E-35	N	13.32	20.16	43.38
AT2G28350	ARF10	0001279	auxin response factor 18-like	0	Y	5.09	5.04	4.42
AT5G61850	LFY	0000040	dna polymerase theta	4.14	NSH	0.001	0.001	0.001
AT5G60690	REV	0001618	homeobox-leucine zipper protein revoluta	0	Y	7.93	9.77	7.39
AT3G44750	HD2A	0027114	histone deacetylase isoform 2	8.55E-30	Y	286.77	270.64	131.72
AT2G45450	ZPR1	0041901	PREDICTED: uncharacterized protein LOC101244019	0.03	N	1.46	1.48	3.71
AT1G55580	LAS	0001820	scarecrow-like protein 28	2.58E-50	N	10.83	13.6	4.89
AT1G79580	SMB	0015551	no apical meristem family protein	1.04E-80	Y	5.84	6.09	3.13
AT1G26870	FEZ	NSH	NSH	NSH	NSH	0.1	0.1	0.1
AT1G56010	NAC1	0016854	nac domain-containing protein 21 22-like	2.92E-70	Y	26.76	33.91	47.81
AT2G04160	AIR3	0002155	subtilisin-like protease-like	0	N	6.59	10.36	37.91
AT3G25710	TMO5	0015249	transcription factor bhlh30-like	1.25E-64	N	7.38	6.47	4.98
AT5G42800	DFR	0031589	dihydroflavonol reductase	4.90E-104	Y	0.38	0.36	1.38
AT4G30080	ARF16	0001279	auxin response factor 18-like	0	Y	5.09	5.04	4.42
AT4G25470	CBF2	0025501	dehydration-responsive element-binding protein	1.90E-45	N	1.11	1.73	1.02
AT1G04240	SHY2 (IAA3)	0014302	auxin-induced protein 22b	1.21E-70	N	35.46	36.07	16.64
AT4G37650	SHR	0003271	protein short-root	5.85E-172	Y	15.86	18.57	12.14
AT5G03150	JKD	0003299	zinc finger protein jackdaw-like isoform x1	3.80E-96	Y	15.2	16.09	12.09
AT3G54220	SCR	0003217	protein scarecrow-like	0	Y	16.24	26.26	18.73
AT1G03840	MGP	0005022	zinc finger protein magpie-like	8.02E-127	Y	9.11	14.27	11.97
AT1G50420	SCL3	0003587	scarecrow-like protein 3	0	Y	12.54	13.71	17.92
AT5G11260	HY5	0015206	transcription factor hy5-like	1.04E-46	Y	49.43	33.1	27.33
AT4G32880	ATHB8	0000849	homeobox-leucine zipper family protein	0	Y	12.14	14.87	11.42
AT4G14550	IAA14	0018103	auxin-induced protein aux28	6.06E-99	Y	343.11	351.74	185.48

AT5G10510	AIL6	0003496	ap2-like ethylene-responsive transcription factor ail6-like	5.36E-115	Y	23.18	27.88	14.55
AT1G19850	MP	0000685	transcriptional factor b3 family protein auxin-responsive factor aux iaa-related	0	Y	5.84	8.35	33.42
AT3G20840	PLT1	0004605	ovule development family protein	0	Y	15.14	23.61	9.93
AT1G04550	IAA12	0009934	auxin-responsive protein iaa11-like	6.86E-58	N	22.94	32.12	39.07
AT1G12980	ESR1	0012720	ethylene-responsive transcription factor 4-like	1.54E-16	N	21.46	33.81	55.6
AT5G61420	MYB28	0035249	transcription factor myb35	3.00E-68	N	0.41	0.73	1.62
AT2G20610	SUR1	0007195	tyrosine aminotransferase-like	7.98E-165	N	27.15	29.93	35.17
AT3G12280	RBR1	0001201	retinoblastoma-related protein	0	Y	18.44	21.99	19.38
AT2G33880	WOX9	0045416	wuschel-related homeobox 11-like	1.43E-22	N	1.15	0.48	0.54
AT1G53230	TCP3	0003653	transcription factor tcp4-like	4.30E-70	N	2.99	3.01	3.47
AT1G19220	ARF19	0000777	auxin response factor 19-like	0	Y	9.5	13.97	12.13
AT2G42430	LBD16	0023003	lob domain-containing protein 16	4.33E-65	Y	1.6	1.25	9.77
AT3G58190	LBD29	0010011	lob domain-containing protein 29-like	9.93E-63	N	3.23	4.69	9.43
AT2G20180	PIL5	0021486	transcription factor pif3-like	1.29E-26	N	0.85	1.33	1.16
AT1G03430	AHP5	0018863	histidine-containing phosphotransfer	2.51E-56	N	47.56	43.54	74.89
AT5G54510	DFL1	0006889	indole-3-acetic acid-amido synthetase	0	Y	22.05	32.66	83.56
AT5G20730	ARF7	0000777	auxin response factor 19-like	0	Y	12.14	14.87	11.42
AT3G15540	IAA19	0015499	auxin-induced protein aux22	3.88E-50	Y	8.92	10.69	8.52
AT3G50340		0010064	PREDICTED: uncharacterized protein LOC100262755	3.12E-165	Y	3.36	4.24	2.85
AT2G45420	LBD18	0044113	lob domain-containing protein 18-like	3.58E-67	N	0.36	0.68	1.73
AT4G14560	IAA1	0014302	auxin-induced protein 22b	6.76E-53	N	35.46	36.07	16.64
AT4G37390	YDK1	0004749	probable indole-3-acetic acid-amido synthetase	0	Y	10.5	18.51	17.66
AT4G27260	WES1	0006889	indole-3-acetic acid-amido synthetase	0	Y	22.05	32.66	83.56

AT5G17430	BBM	0002613	ap2-like ethylene-responsive transcription factor bbm-like	1.34E-122	Y	9.55	13.02	6.82
AT5G03740	HD2C	0009674	histone deacetylase hdt1-like	1.02E-24	N	139.84	161.42	92.97
AT5G22650	HD2B	0027114	histone deacetylase isoform 3	8.21E-26	N	286.77	270.64	131.72
AT5G18560	PUCHI	0012720	ethylene-responsive transcription factor 4-like	3.71E-23	N	21.46	33.81	55.6
AT5G37020	ARF8	0001138	auxin response factor 8-like	0	Y	9.71	9.76	5.8
AT1G28130	GH3	0004939	indole-3-acetic acid-amido synthetase -like	0	Y	7.5	7.25	14.31
AT5G60890	MYB34	0035249	transcription factor myb34	1.30E-67	N	0.41	0.73	1.62
AT4G00220	LBD30	0044113	lob domain-containing protein 18-like	5.91E-59	Y	0.36	0.68	1.73
AT2G01420	PIN4	0002917	auxin efflux carrier component 3-like	0	Y	1.66	2.17	5.65
AT1G70940	PIN3	0002917	auxin efflux carrier component 3-like	0	Y	1.66	2.17	5.65
AT1G23080	PIN7	0002917	auxin efflux carrier component 3-like	0	Y	1.66	2.17	5.65

Supplementary Table S7. List of primers used in this work.

Primer Name	Sequence
PpUBC9_fw	GGTGTTCCAGGTATTGAGCTG
PpUBC9_rv	AGCCATCCTCACAAAAGCAACC
PpYLS8_fw	ACCGTTCAGGTTGAGGCGAAG
PpYLS8_rv	GATACCCCAAACTAAGCATAGGC
PpEF1a_fw	GAGGTCTGAAGTTATGGGTCGTG
PpEF1a_rv	AGCACACCGACGCTACTAATG
* PpTFB4_fw	GCAATGCCTATAGCTCCTCCG
* PpTFB4_rv	GTTGTTGCCCGAGTCACCTTG
* PpHD2C_fw	GGTGGAAAGACTCCTGGTGGAG
* PpHD2C_rv	GTGCTTGGCCTTTGTGTGAGAC
* PpXPL1_fw	GGCCAGATGCTTAGAGATGCTG
* PpXPL1_rv	CTCGGATGAACTGATCAGTCC
* PpFBR_fw	CTGCAGCAAGACCAGTTCAAGC
* PpFBR_rv	CTGCTTCTTAGGCATGCGGTAAC
* PpGASA_fw	GCTCTCATTGCCATTTCCATGC
* PpGASA_rv	GAAGGCTTGACGCTGCCAGG
* PpHD2A_fw	GGTTTTGGAGTCTAGCGAGGAATG
* PpHD2A_rv	TTCCCTGTCTGCCTGTTCTGTTTC
PpSUR1_fw	TGTTAGATGTCACCCGTTGCTG
PpSUR1_rv	CATGACCATTGTGCCTGATTAGC
PpZIP9_fw	TCTGAACTGTGGTCTTGGGAATC
PpZIP9_rv	TCACAATCAACTCAACTAGCACC
PpIAA14_fw	CTGGGCAAGATGTTCAGTTCCT
PpIAA14_rv	AAGATCAGCGGAACAACATCAGC
PpGH3_fw	TCAACCAAACACCCCAAAGAGC
PpGH3_rv	TCAACCAAACACCCCAAAGAGC

* primers reported in Shishkova *et al.* (2013), designed for regions with 100% homology between *Stenocereus gummosus* and *Pachycereus pringlei* genes.
